# Soluble TIM3 and Its Ligands Galectin-9 and CEACAM1 Are in Disequilibrium During Alcohol-Related Liver Disease and Promote Impairment of Anti-bacterial Immunity

**DOI:** 10.3389/fphys.2021.632502

**Published:** 2021-03-10

**Authors:** Antonio Riva, Elena Palma, Dhruti Devshi, Douglas Corrigall, Huyen Adams, Nigel Heaton, Krishna Menon, Melissa Preziosi, Ane Zamalloa, Rosa Miquel, Jennifer M. Ryan, Gavin Wright, Sarah Fairclough, Alexander Evans, Debbie Shawcross, Robert Schierwagen, Sabine Klein, Frank E. Uschner, Michael Praktiknjo, Krum Katzarov, Tanya Hadzhiolova, Slava Pavlova, Marieta Simonova, Jonel Trebicka, Roger Williams, Shilpa Chokshi

**Affiliations:** ^1^Institute of Hepatology, Foundation for Liver Research, London, United Kingdom; ^2^Faculty of Life Sciences & Medicine, King’s College London, London, United Kingdom; ^3^Department of Gastroenterology, Basildon University Hospital, Basildon, United Kingdom; ^4^Department of Gastroenterology, Royal Berkshire Hospital, Reading, United Kingdom; ^5^Institute of Liver Studies, King’s College London, London, United Kingdom; ^6^Liver Histopathology Laboratory, Institute of Liver Studies, King’s College Hospital, London, United Kingdom; ^7^Gastrointestinal and Liver Services, Royal Free Hospital, London, United Kingdom; ^8^Translational Hepatology, Department of Internal Medicine I, University Hospital Frankfurt, Frankfurt, Germany; ^9^Department of Internal Medicine I, University of Bonn, Bonn, Germany; ^10^Department of Gastroenterology, Hepatobiliary Surgery and Transplantology, Military Medical Academy, Sofia, Bulgaria; ^11^European Foundation for the Study of Chronic Liver Failure (EF-CLIF), Barcelona, Spain

**Keywords:** TIM3, immune checkpoint, alcohol, biomarker, alcohol-related liver disease

## Abstract

**Background and Aims:**

Immunoregulatory checkpoint receptors (CR) contribute to the profound immunoparesis observed in alcohol-related liver disease (ALD) and *in vitro* neutralization of inhibitory-CRs TIM3/PD1 on anti-bacterial T-cells can rescue innate and adaptive anti-bacterial immunity. Recently described soluble-CR forms can modulate immunity in inflammatory conditions, but the contributions of soluble-TIM3 and soluble-PD1 and other soluble-CRs to immune derangements in ALD remain unclear.

**Methods:**

In Alcoholic Hepatitis (AH; *n* = 19), alcohol-related cirrhosis (ARC; *n* = 53) and healthy control (HC; *n* = 27) subjects, we measured by Luminex technology (i) plasma levels of 16 soluble-CRs, 12 pro/anti-inflammatory cytokines and markers of gut bacterial translocation; (ii) pre-hepatic, post-hepatic and non-hepatic soluble-CR plasma levels in ARC patients undergoing TIPS; (iii) soluble-CRs production from ethanol-treated immunocompetent precision cut human liver slices (PCLS); (iv) whole-blood soluble-CR expression upon bacterial challenge. By FACS, we assessed the relationship between soluble-TIM3 and membrane-TIM3 and rescue of immunity in bacterial-challenged PBMCs.

**Results:**

Soluble-TIM3 was the dominant plasma soluble-CR in ALD vs. HC (*p* = 0.00002) and multivariate analysis identified it as the main driver of differences between groups. Soluble-CRs were strongly correlated with pro-inflammatory cytokines, gut bacterial translocation markers and clinical indices of disease severity. Ethanol exposure or bacterial challenge did not induce soluble-TIM3 production from PCLS nor from whole-blood. Bacterial challenge prompted membrane-TIM3 hyperexpression on PBMCs from ALD patient’s vs. HC (*p* < 0.002) and was inversely correlated with plasma soluble-TIM3 levels in matched patients. TIM3 ligands soluble-Galectin-9 and soluble-CEACAM1 were elevated in ALD plasma (AH > ARC; *p* < 0.002). *In vitro* neutralization of Galectin-9 and soluble-CEACAM1 improved the defective anti-bacterial and anti-inflammatory cytokine production from *E. coli*-challenged PBMCs in ALD patients.

**Conclusions:**

Alcohol-related liver disease patients exhibit supra-physiological plasma levels of soluble-TIM3, particularly those with greater disease severity. This is also associated with increased levels of soluble TIM3-ligands and membrane-TIM3 expression on immune cells. Soluble-TIM3 can block the TIM3-ligand synapse and improve anti-bacterial immunity; however, the increased levels of soluble TIM3-binding ligands in patients with ALD negate any potential immunostimulatory effects. We believe that anti-TIM3 neutralizing antibodies currently in Phase I clinical trials or soluble-TIM3 should be investigated further for their ability to enhance anti-bacterial immunity. These agents could potentially represent an innovative immune-based supportive approach to rescue anti-bacterial defenses in ALD patients.

## Introduction

Alcohol-related liver disease (ALD) represents a significant public health burden, and according to the World Health Organization alcohol is “the third highest risk factor for premature mortality, disability and loss of health worldwide” (Soria Saucedo, 2013). Whilst ALD encompasses a spectrum of clinical manifestations, it is well recognized that advanced disease is associated with multiple derangements in host immunity and one of the major and most common complications that patients face is an increased vulnerability to bacterial infection, which can lead to worsening of disease and multi-organ failure (Louvet and Mathurin, 2015; Albillos et al., 2014; EASL, 2018).

Patients with alcohol-related cirrhosis (ARC) are highly susceptible to overwhelming bacterial infections, which increases their probability of death by 3.75-fold, reaching 30% at 1-month and 63% at 1-year (Albillos et al., 2014; Gustot et al., 2014; Jalan et al., 2014; Louvet and Mathurin, 2015). In patients with alcoholic hepatitis (AH), the most florid form of ALD, the susceptibility to infection is further heightened and is the leading cause of death, with infection observed in up to 65% of cases (Louvet et al., 2009, 2015). It is also the commonest precipitating event for acute-on-chronic liver failure (ACLF) (Arroyo et al., 2015; Trebicka et al., 2020).

The disease state in advanced ALD represents an immunological paradox. Patients exhibit a multi-systemic hyperactivated immunity at the clinical and molecular level, which can co-exist with immune inactivation. This landscape, which is progressively established during ARC and is a hallmark of AH (Mathurin and Lucey, 2012; Albillos et al., 2014; Louvet and Mathurin, 2015; Markwick et al., 2015; EASL, 2018), underlies the rampant inflammation and profound predisposition to bacterial infection (Mathurin and Lucey, 2012; Louvet and Mathurin, 2015; EASL, 2018). To date, therapeutics have primarily focussed on (i) curtailing the infection with use of widespread antibiotics, concerningly this has promoted development of multi-drug resistant microbes (Merli et al., 2015; Fernandez et al., 2016; EASL, 2018), or (ii) bridling the rampant inflammation with immunosuppressive agents. However, this latter strategy can potentiate the immunocompromised state and increase the risk of secondary infections (Louvet et al., 2009; Thursz et al., 2015; Vergis et al., 2017; Chokshi, 2018). Targeted immunomodulatory approaches to restore the disrupted balance between protective anti-pathogen immunity and host-induced immunopathology are lacking.

Preservation of this homeostatic equilibrium physiologically is achieved through multi-faceted immunoregulatory networks and a major ‘tenet’ are checkpoint receptors (CRs), which activate or inhibit immune cells in a temporal and anatomically coordinated manner (Riva and Chokshi, 2018). Best known for their involvement in suppressing anti-tumor immunity, blockade with neutralizing antibodies to PD1 has obtained FDA approval in multiple cancers including hepatocellular carcinoma. Pre-clinical and clinical studies have also described a role for checkpoint receptors including PD1 and TIM3 in sepsis (Patil et al., 2017) and septic shock, where increased membrane-bound immune cell expression has been associated with a higher rate of nosocomial infections and mortality (Guignant et al., 2011). Of note, a recent phase-1b randomized controlled trial of checkpoint inhibitor therapy in 31 immunocompromised patients with sepsis (Hotchkiss et al., 2019b) found it well tolerated with no evidence of treatment-related hypercytokinemia. Further to this, a second study assessing the utility of anti-PDL1 in sepsis was also well tolerated and at higher doses there was evidence of immune restoration (Hotchkiss et al., 2019a).

We were the first to demonstrate that membrane-bound PD1 and membrane-bound TIM3 on T-cells impair their anti-bacterial functionality in AH (Markwick et al., 2015). Moreover, we showed that *ex vivo* blockade using neutralizing antibodies led to reconstitution of both innate and adaptive arms of the anti-bacterial immunity without exacerbating the production of cytokines associated with systemic inflammation. However, the individual contributions of these two membrane-bound checkpoints remained unclear. Furthermore, while CRs were initially discovered as membrane-bound molecules (membrane-CRs), we now know that many can exist in soluble form (soluble-CRs), generated by alternative mRNA splicing or metalloprotease-mediated ectodomain shedding (Gu et al., 2018; Riva and Chokshi, 2018). These soluble-CRs can act as agonists or antagonistic molecular decoys and can orchestrate host immunity distally, performing paracrine tasks similar to stimulatory or inhibitory cytokines. Systemic concentrations of several soluble-CRs rise during inflammation, autoimmunity (Jung et al., 2003; Lahat et al., 2003; Ip et al., 2005, 2006; Cao et al., 2012; Delmastro et al., 2012; Chiba et al., 2017; Zhao D. et al., 2017; Lin et al., 2018), infectious diseases (Cao et al., 2011; Clayton et al., 2015; Ren et al., 2015; Zilber et al., 2019), and cancer (Prigent et al., 1999; Triebel et al., 2006; Hock et al., 2009; Heo et al., 2012; Ge et al., 2017; Silva et al., 2017; Zhao Q. et al., 2017; He et al., 2018; Li N. et al., 2018) often mirroring immune dysfunction, disease progression and increased mortality. Measurements of systemic soluble-CRs are useful both as potential diagnostic/prognostic biomarkers (Chen et al., 2017; Li Y. M. et al., 2018) but also to expose mechanisms underlying immunopathogenesis of disease. The contribution of soluble-CRs in ALD, particularly soluble-PD1 and soluble-TIM3, remains unclear and defining it was the aim of this investigation.

We report that the soluble-TIM3/ligand axis is significantly dysregulated in ALD, whereas the soluble-PD1 pathway does not seem to be involved. We show that soluble-TIM3 and both its soluble ligands Galectin-9 and CEACAM-1 were significantly elevated in the plasma of ALD patients. Interestingly, we show that unlike the membrane-bound form, the soluble-TIM3 pathway is immunostimulatory. However, we suggest that in the context of ALD, the immune potentiating properties of this pathway may be hampered by the high levels of ligand-receptor neutralisation in the systemic circulation.

## Materials/Patients and Methods

### Subjects and Samples

The study was performed conforming to the declaration of Helsinki, with full informed patient consent and ethical approval from all recruiting centers (United Kingdom Research Ethics Committee reference numbers 13/SW/0219, 08/H0702/52 and 12/SC/0359; Bulgarian Ethics Protocol 1/27.02.18). We included: AH, *n* = 19, with Maddrey’s discriminant function ≥32 (Maddrey et al., 1978), excluding patients receiving immunosuppressants prior-to/at-time-of sampling; Compensated/decompensated ARC, *n* = 33, excess active alcohol drinkers (>60g/>80g female/male per day) seen as out-patients, excluding patients with cancer, gastrointestinal bleeding, untreated sepsis, or immunomodulatory treatments; Healthy volunteers as healthy controls (HC, *n* = 27).

In sub-groups of patients we assessed: (i) soluble-CRs in plasma and in whole-blood or peripheral blood mononuclear cell (PBMC) cultures challenged with *Escherichia coli*; (ii) membrane-CRs on PBMCs, challenged with *E. coli* as previously described (Riva et al., 2018); (iii) *in vitro* biological activity of soluble-TIM3 in PBMCs challenged with *E. coli* and treated with/without recombinant soluble-TIM3. Soluble-CR levels were also measured (iv) in whole-blood plasma obtained from four anatomical sites (portal/hepatic/cubital vein, right cardiac atrium) from 20 decompensated ARC patients during Transjugular Intrahepatic Portosystemic Shunt (TIPS) procedure (ascites = 17; varices = 3) (Bonn University Ethics Committee reference number 029/13) and (v) in a novel human organotypic liver culture model of acute ethanol exposure (precision-cut liver slices, PCLS). PCLS were prepared from the healthy (tumor-free) portion of liver resections from 3 patients undergoing partial hepatectomy for colorectal liver metastases (fibrosis score F1–F2, *n* = 2; F2–F3, *n* = 1) and 1 patient undergoing partial hepatectomy for adrenocortical carcinoma liver metastases (United Kingdom Research Ethics Committee reference number 17/NE/0340, IRAS ID222302) as previously described (Palma et al., 2020). Table 1 summarizes the clinical characteristics of patients.

**TABLE 1:** Baseline patient characteristics.

	***p*-value**	**HC**	**ARC**	**SAH**	**PCLS**	**TIPS**
Gender (M/F/na)*		10/15/2	23/10/0	13/6/0	1/3/0	12/8/0
Age (years)	7.3E-10	32.00	56.00	47.00	60.00	61.00
		(26, 41)	(49, 64)	(41, 52.5)	(46.5–73.5)	(53.75–66.00)
Child-Pugh category (A/B/C)*		–	8/12/12	–	–	1/15/4
Child-pugh score	4.1E-3	–	9.0	11.0	–	8
			(6.75, 10)	(10, 12)		(7–9)
MELD score	3.0E-4	–	13.67	21.41	–	15.00
			(8, 20.33)	(19.87, 26.98)		(10.50–18.25)
Maddrey’s discriminant function		–	–	44.19	–	–
				(37.96, 58.1)		
Creatinine (μmol/L)		–	70.00	71.00	81.00	119.34
			(56.5, 101)	(60.5, 87.5)	(62.5–103.5)	(91.94–145.86)
Total bilirubin (μmol/L)	1.6E-7	–	34.00	251.00	6.00	24.62
			(19, 62)	(146, 369)	(3–10.5)	(15.9–45.14)
AST (IU/L)	7.1E-4	–	54.50	115.00	26.50	51.00
			(43.5, 65.75)	(92, 234)	(24.25–78.25)	(26.05–72.50)
GGT (IU/L)		–	127.50	341.00	–	166.50
			(93, 208)	(116, 679)		(96.00–265.25)
ALP (IU/L)	8.3E-3	–	140.50	248.00	97.50	135.00
			(95.25, 177.5)	(149, 312.5)	(94.75–99)	(104.75–149.75)
Albumin (g/dL)	6.1E-3	–	33.00	28.00	4.00	31.30
			(27, 38)	(24.5, 32)	(3.4–4.15)	(25.90–35.80)
INR		–	1.50	1.60	1.02	1.20
			(1.2, 1.7)	(1.5, 1.85)	(0.99–1.06)	(1.10–1.43)
Sodium (mmol/L)	4.2E-2	–	135.50	133.00	139.50	138.00
			(133.75, 140)	(132.5, 135)	(138.5–140.25)	(136.00–139.75)
Platelets (billion/L)		–	114.00	117.00	241.50	165.00
			(73, 173)	(83, 182.5)	(237.25–259)	(114.50–221.50)
Total leukocytes (billion/L)	2.2E-5	–	5.90	11.10	6.26	7.96
			(4.21, 7.87)	(8.1, 16.95)	(5.75–6.95)	(6.51–10.51)
Neutrophils (billion/L)	2.8E-5	–	3.46	9.71	3.79	5.65
			(2.21, 4.9)	(5.77, 12.21)	(3.63–4.18)	(3.90–7.46)
Lymphocytes (billion/L)		–	1.29	1.59	1.60	1.60
			(0.86, 1.65)	(1.11, 2.18)	(1.49–1.96)	(1.10–2.04)
Monocytes (billion/L)	7.7E-5	–	0.42	1.00	0.35	0.92
			(0.3, 0.6)	(0.84, 1.3)	(0.29–0.43)	(0.66–1.40)
Neutrophil-to-lymphocyte ratio (NLR)	3.5E-3	–	2.64	6.48	2.24	3.66
			(1.65, 3.8)	(3.12, 7.99)	(1.96–2.82)	(2.2–4.93)
Monocyte-to-lymphocyte ratio (MLR)	5.0E-3	–	0.34	0.57	0.18	0.63
			(0.24, 0.48)	(0.42, 0.76)	(0.12–0.3)	(0.35–0.77)

*Absolute counts.All other parameters = median (IQR).ALP, alkaline phosphatase; ARC, alcohol-related cirrhosis; AST, aspartate transaminase; GGT, gamma-glutamyl transpeptidase; HC, healthy control; INR, international normalized ratio; MELD, model for end-stage liver disease; SAH, severe alcoholic hepatitis; TOT, total number.

### Preparation of Fixed *Escherichia coli*

*Escherichia coli* (*E. coli*) DH5-alpha was prepared as previously described (Riva et al., 2018). Briefly, *E. coli* batches were grown in RPMI 1640 medium, fixed in BD Cytofix buffer (formaldehyde 4% in PBS, BD-Biosciences, United Kingdom) for 10 min at room temperature, extensively washed in PBS (Gibco/Thermo Fisher Scientific, United Kingdom) and stored at 4–8°C (or −80°C for long-term storage). Bacterial concentrations were determined by visual counting with a Neubauer-Petroff 0.02 mm chamber.

### Soluble-CR and Cytokine Production From Whole-Blood Cultures Challenged With *E. coli*

Fresh whole-blood cultures were established within 2 h of collection. Briefly, whole blood was cultured at 37°C, 5% CO_2_ in round-bottom cell-culture tubes for 2 h, with or without stimulation with *E. coli* at 10 bacteria-per-cell (BpC); supernatants were cryopreserved at −80°C for Luminex analysis of soluble-CR and cytokine production.

### Soluble-CR Production From a Human Organotypic Liver Culture Model of Acute Ethanol Exposure

An immunocompetent human precision-cut liver slice (PCLS) model of acute ethanol exposure was utilized to assess soluble-CR production in response to ethanol. Slices were prepared according to established protocols (Palma et al., 2019, 2020). After 2 h of recovery, slices were treated with ethanol 250 mmol/L for 24 h. At the end of culture, slices were harvested for haematoxylin/eosin staining. PCLS culture supernatants were cryopreserved at −80°C for Luminex analysis. To assess ethanol treatment toxicity, cytokeratin (CK) 18 fragments, surrogate markers of cell toxicity and apoptotic cell death, were measured in PCLS supernatants by ELISA according to manufacturer’s guidelines (Peviva, M65 EPIDEATH^®^ ELISA and M30 CYTODEATH^TM^ ELISA).

### Temporal Relationship Between Membrane- and Soluble-CR Production From PBMCs Challenged With *E. coli*

PBMCs were isolated and cryopreserved according to well established protocols (Riva et al., 2014). PBMCs were defrosted, counted and cultured at 37°C, 5% CO_2_, in round-bottom 96-well plates (300,000/200 μL/well) in complete supplemented (s)RPMI medium [RPMI 1640, 100 IU/mL Penicillin/Streptomycin, 2.2 mmol/L L-glutamine, 23mmol/L HEPES (Gibco/Thermo Fisher Scientific, United Kingdom)] with 10% human AB serum (Sigma-Aldrich/Merck, United Kingdom). Cell viability was determined with an automated ADAM cell counter. For membrane-CR/soluble-CR kinetics, PBMCs from HC, ARC and SAH (*n* = 10/10/10) were unstimulated/stimulated with *E. coli* (10 BpC) in replicates for 7 days. Daily supernatants were collected and cryopreserved at −80°C for soluble-CR Luminex analysis. On days 1, 4, and 7 PBMCs were collected for flow cytometric evaluation of membrane-CR expression. Briefly, cells were pelleted, resuspended in PBS and stained with Ghost Dye^TM^ Red 710 (Tonbo Biosciences/Cambridge Bioscience, Cambridge, United Kingdom) for live/dead discrimination. Cells were then washed, resuspended in FACS buffer (PBS without Ca^2+^/Mg^2+^, 1% FBS) and stained with antibodies (20 min, 4°C) to: CD3, CD4, CD8, CD14, CD19, CD56, CD16, and TIM3, then washed and fixed with BD Cytofix buffer for analysis.

Functional assessments were conducted by blocking the interaction between TIM3 and its ligands using alpha-lactose 30 mmol/L (L2643, Sigma-Aldrich/Merck, United Kingdom) and/or recombinant soluble TIM3-Ig fusion protein at 5 μg/mL (rhsTIM3-Ig, 2365-TM, Bio-Techne, United Kingdom) cultured with PBMCs from HC and SAH (*n* = 5/5) for 1 h. When in combination, alpha-lactose was administered for 1 h, followed by soluble-TIM3 for another hour. Subsequently, *E. coli* was added for 1 h and intracellular transports were blocked with Brefeldin-A 10 μg/mL (B7651, Sigma-Aldrich/Merck, United Kingdom) during the last overnight incubation. Cells were incubated overnight, resuspended in FACS buffer and stained for intracellular flow cytometry as previously described (Riva et al., 2018) for CD3, CD4 and CD8 and with intracellular antibodies for IFNγ, IL-17A and IL-10.

Specific Galectin-9 neutralization was subsequently assessed in PBMC cultures from SAH patients (*n* = 4) blocked with a monoclonal neutralizing anti-Galectin-9 antibody at 10 μg/mL (MABT834, clone 9S2-1, Sigma-Aldrich/Merck, United Kingdom) for 1 h. *E. coli* was added for 1 h and intracellular transports were blocked with Brefeldin-A 10 μg/mL (B7651, Sigma-Aldrich/Merck, United Kingdom). Cells were incubated for up to 48 h, resuspended in FACS buffer and stained for intracellular flow cytometry as previously described (Riva et al., 2018) for CD3, CD4, CD8, and CD14 and with intracellular antibodies for IFNγ, IL-17A and IL-10.

For antibody clones and fluorochromes see Supplementary Table 1.

### Multiplex Quantification of Soluble-CRs, Cytokines and Bacterial Translocation Markers

Soluble-CRs were quantified by Luminex following manufacturer’s instructions, using a MAGPix instrument with xPonent v4.2 software (LuminexCorp, ’s-Hertogenbosch, Netherlands). Soluble-CRs included TIM3, CD80, LAG3, HVEM, BTLA, CD27, CD28, CD137, CD152, GITR, IDO, PDL1, PDL2, and PD1) (Thermo Fisher Scientific, United Kingdom). Using the same platform, we also measured TIM3 soluble ligands Galectin-9 and soluble-CEACAM1, 10 pro-/anti-inflammatory cytokines (IL-1a, IL-1b, IL-1Ra, IL-6, IL-8, IL-10, IL-18, IL-33, IFNg, and TNFa), and the gut bacterial translocation marker soluble-CD163 (R&D-Systems/Bio-Techne, Abingdon-Oxford, United Kingdom). Another gut bacterial translocation marker, D-lactate, was measured by colorimetric assay (AbCam, Cambridge, United Kingdom).

### Statistical Analyses

We used: Mann–Whitney (MW) test or Kruskal–Wallis (KW) test with Dunn’s *post hoc* correction for independent samples; Wilcoxon Signed Rank test or Repeated-Measures Two-way ANOVA with Holm–Sidak’s *post hoc* correction for paired samples; Mixed Model analysis for time-dependent cell-culture kinetics; Chi-square test for categorical variables; Pearson’s *R* or Spearman’s r correlation coefficients as appropriate. Multiple hypothesis testing adjustments were: Benjamini–Hochberg false discovery rate (BH, FDR) for group comparisons; Bonferroni family-wise error (FWER) for correlations. Group differences for multiplex assays were considered ‘significant’ with *p* ≤ BH-threshold or ‘trends’ with BH-threshold < *p* ≤ 0.05. Multicollinearity was investigated by correlation matrix analysis (bivariate) and Variance Inflation Factor (VIF) regression diagnostics (multivariate). Basic statistics were calculated with IBM SPSS 25 (Armonk, NY, United States), GraphPad Prism 8 (San Diego, CA, United States) and Microsoft Excel 2016 (Redmond, WA, United States). Correlation heatmaps were produced with RStudio 1.3.959, R 5.3.2 and the package ‘corrplot’ (Wei and Simko, 2017; R_Core_Team, 2020; RStudio_Team, 2020).

Multivariate analysis (MVA) was performed by Principal Component Analysis (PCA, unsupervised) and Partial Least Squares (Projection-to-Latent-Structures) discriminant analysis (PLS-DA, supervised). Data for MVA were Log10-transformed, mean-centered and unit-variance scaled. Goodness-of-fit and internal predictive performance were assessed by cumulative R2X/R2Y and Q2, respectively. Model validation and significance were measured by cross-validated residuals ANOVA (CV-ANOVA) and misclassification tables. MVA was performed with Umetrics SIMCA 15 (Umeå/Malmö, Sweden).

Statistical significance, FWER and FDR were all set at two-tailed alpha = 5.0E-2.

## Results

### Patients Characteristics

As summarized in Table 1, AH patients were significantly sicker than ARC, having higher Child-Pugh score, MELD score and INR, increased concentrations of bilirubin, AST, ALP and GGT and lower albumin levels. AH patients also had higher leukocyte count (particularly neutrophils and monocytes), neutrophil-to-lymphocyte ratio (NLR) and monocyte-to-lymphocyte ratio (MLR) compared to ARC patients. ALD patients were older than HC and among ARC patients, those with Child-Pugh A were younger.

### Soluble-TIM3 Is Severely Dysregulated in ALD

**FIGURE 1:**
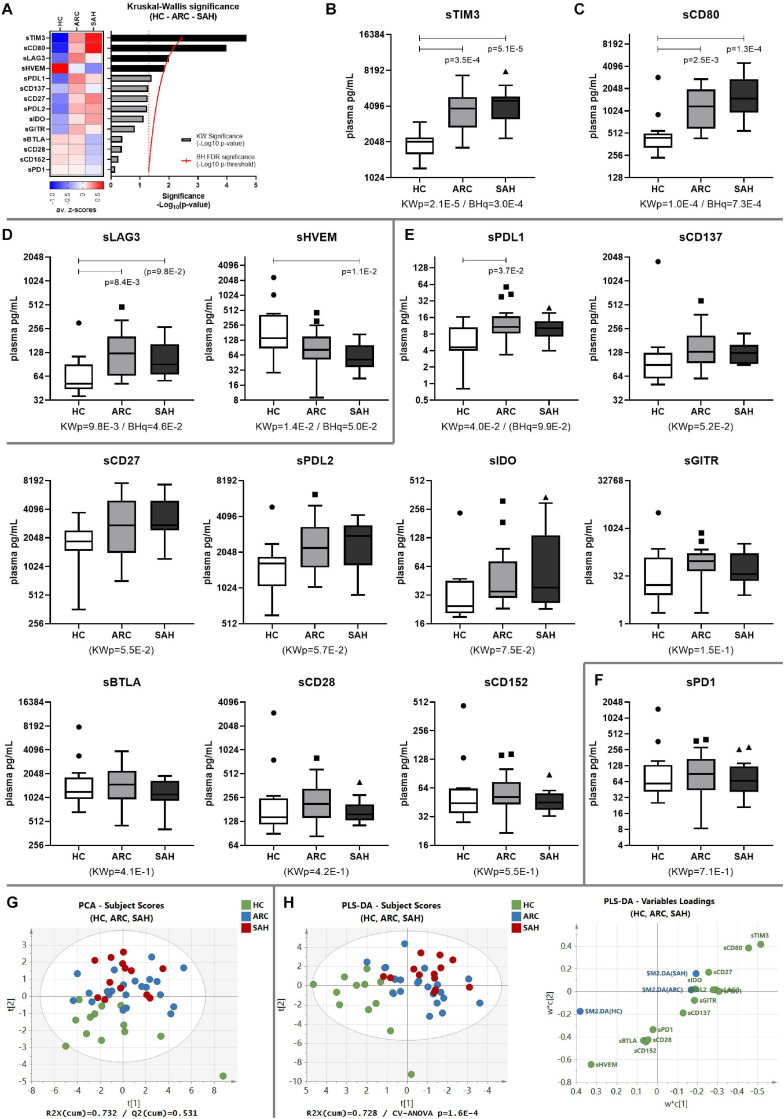
Soluble-CR networks in ALD patients. **(A)** Significance plot summarizing the soluble-CR differential expression in HC, ARC and SAH; soluble-CR measurements: standardized *z*-scores; significance: –Log_10_(*p*-value); red continuous line: BH significance threshold; red dotted line: *p* = 0.05; black bars: significantly different soluble-CRs; gray bars: non-significant soluble-CRs. **(B)** Soluble-TIM3 is the most different and highly upregulated soluble-CR in patients vs. HC, followed by **(C)** soluble-CD80, **(D)** soluble-LAG3 and soluble-HVEM. **(E)** Soluble-CRs comparable between groups. **(F)** Soluble-PD1 is the most similar in patients and controls. KWp, raw Kruskal–Wallis *p*-value; BHq, FDR-adjusted *q*-value; p, significant multiple comparisons with Dunn’s correction. Boxplots (median, IQR, ± Tukey’s whiskers/outliers) ordered by decreasing statistical significance. **(G)** Subject clustering by unsupervised PCA. **(H)** 3-way supervised clustering by PLS-DA, with superimposition of HC, ARC, and SAH categories; soluble-TIM3 is the main driver of supervised clustering, as shown in the ‘Variables Loadings’ plot; subject re-classification based on this model: 70.8% accuracy (HC-ARC-SAH = 100%–86.4%–15.4%, Fisher’s *p* = 8.7E-9). ‘Subject Scores’ plots: each dot represents a subject; ‘Variables Loadings’ plots: each dot represents a soluble-CR.

Systemic levels of soluble-TIM3 were highly elevated in ALD patients and the most significantly different between groups amongst all the soluble-CRs (Supplementary Table 2 and Figures 1A,B). Increased levels of soluble-CD80 were also observed (ALD > HC, Figures 1A,C). Compared to HC, soluble-LAG3 was also greater in patients (ALD > HC), while soluble-HVEM was lower in patients (ALD < HC); however, differences for soluble-LAG3 and soluble-HVEM between groups were only borderline significant (Figures 1A,D). All other soluble-CRs were comparable between patients and controls (Figures 1A,E), and interestingly soluble-PD1 was the least different amongst all (Figures 1A,F).

To investigate the combined effect of multiple soluble-CRs on the performance of soluble-TIM3 as a disease marker, multivariate analysis of plasma soluble-CRs was performed in the three study groups. Principal component analysis (PCA) highlighted a degree of unsupervised internal clustering linked to differential soluble-CR plasma levels (Figure 1G). Superimposition of clinical categories (HC, ARC, and SAH) by partial least squares discriminant analysis (PLS-DA) indicated soluble-TIM3 as the strongest driver of these differences (Figure 1H).

### Soluble-TIM3 and Its Ligands Correlate With ALD Severity and Inflammation

The relationship between plasma soluble-TIM3, other soluble-CRs and indices of inflammation and disease severity was then assessed by correlation analysis.

**FIGURE 2:**
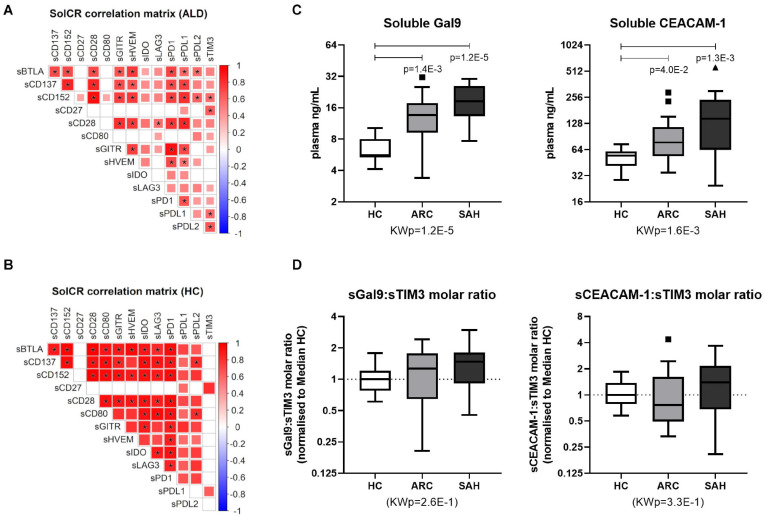
Soluble-TIM3 intercorrelations and soluble-TIM3 ligands. **(A)** Intercorrelation matrix of individual soluble-CRs in ALD patients and **(B)** controls; the color scale and the size of individual matrix squares represent the value of significant Pearson correlation coefficients (*p* ≤ 0.05); blank squares indicate lack of significant correlation; starred squares indicate significant correlations by Bonferroni correction. **(C)** Plasma levels of TIM3 soluble ligands Galectin-9 and soluble-CEACAM1 in HC, ARC and SAH. **(D)** Molar ratios between plasma Galectin-9 or soluble-CEACAM1 and soluble-TIM3 in HC, ARC and SAH, normalized vs. median (HC) (Kruskal–Wallis test with Dunn’s multiple comparison correction).

**FIGURE 3:**
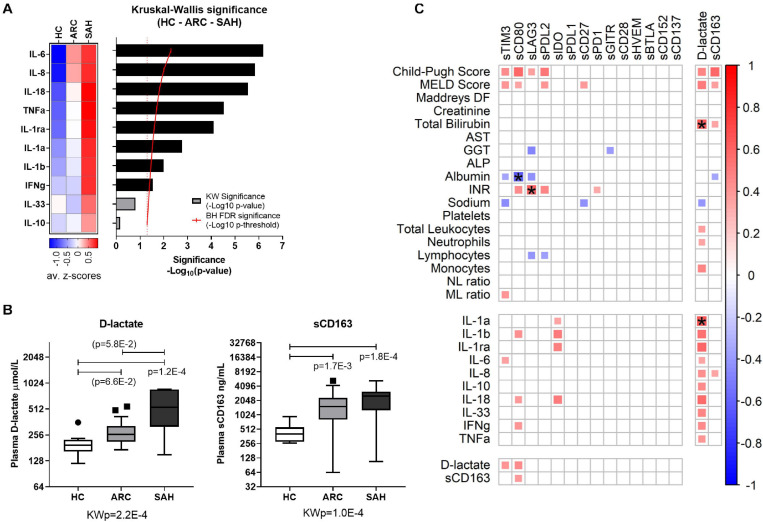
Inflammatory cytokines, bacterial translocation and overall association with the hyper-inflamed state in ALD patients. **(A)** Significance plot summarizing cytokine differential expression in HC, ARC, and SAH; cytokine measurements: standardized *z*-scores; significance: –Log_10_(*p*-value); red continuous line: BH significance threshold; red dotted line: *p* = 0.05; black bars: significantly different cytokines; gray bars: non-significant cytokines. **(B)** Boxplot representation of D-lactate and soluble-CD163 as surrogate markers of bacterial translocation, indicating higher levels in ALD patients compared to HC. KWp, Kruskal–Wallis *p*-value; p, significant multiple comparisons with Dunn’s correction. Boxplots (median, IQR, ± Tukey’s whiskers/outliers). **(C)** Correlation matrix of individual soluble-CRs with clinical parameters, cytokine measurements and markers of bacterial translocation in ALD patients; the color scale and the size of the individual matrix squares represent the value of significant Pearson correlation coefficients (*p* ≤ 0.05); blank squares indicate lack of significant correlation; starred squares indicate significant correlations by Bonferroni correction.

Soluble-CRs were strongly positively intercorrelated with each other in ALD patients (Figure 2A and Supplementary Table 3) and in controls (Figure 2B and Supplementary Table 4). However, soluble-TIM3 and soluble-CD80 correlations were peculiar: the majority of soluble-TIM3 intercorrelations were only present in patients, while most soluble-CD80 intercorrelations were only present in controls. Some multivariate collinearity was also detected by Variance Inflation Factor (VIF) regression diagnostics (Supplementary Table 5). Of note, plasma levels for both TIM3 soluble ligands Galectin-9 and soluble-CEACAM1 were higher in ALD patients (SAH > ARC) compared to HC (Figure 2C). To estimate receptor-ligand binding, the normalized molar ratios for each soluble ligand and soluble-TIM3 were calculated, and for both Galectin-9 and soluble-CEACAM1 these values were comparable across groups (Figure 2D), suggesting a disease-independent molar (quantitative) equilibrium. In ALD patients, the top-three hyper-expressed soluble-CRs displayed several positive correlations with Child-Pugh score (soluble-TIM3, soluble-CD80, and soluble-LAG3), MELD score (soluble-TIM3 and soluble-CD80), INR (soluble-CD80 and soluble-LAG3) and negative correlations with albumin (soluble-TIM3, soluble-CD80, and soluble-LAG3), GGT (soluble-LAG3), sodium (soluble-TIM3) and lymphocyte count (soluble-LAG3) (Figure 3C and Supplementary Table 6). Soluble-TIM3 was also the only soluble-CR positively correlated with the monocyte-to-lymphocyte ratio (MLR), a surrogate index of inflammation (Figure 3C and Supplementary Table 6).

As expected, elevated plasma levels of pro-inflammatory IL-1a, IL-1b, IL-1ra, IL-6, IL-8, IL-18, TNFa, and IFNg were observed in ALD compared to HC (Figure 3A and Supplementary Figure 1). By contrast, IL-10 and IL-33 were comparable between groups. Soluble-TIM3 was the only soluble-CR correlated with the archetypal pro-inflammatory cytokine IL-6 in ALD patients (Figure 3C and Supplementary Table 6). Other cytokine correlations were observed for soluble-CD80 (IL-1b, IL-18, and IFNg) and soluble-IDO (IL-1a, IL-1b, IL-1ra, and IL-18) (Figure 3C and Supplementary Table 6). Of note, no correlations were found in HC between soluble-CRs and cytokines (Supplementary Table 7).

As expected, plasma concentrations of both D-lactate and soluble-CD163, two surrogate markers of gut permeability and bacterial translocation, (i) were significantly higher in ALD patients (SAH > ARC) compared to HC (Figure 3B) reconfirming our previous findings (Markwick et al., 2015; Riva et al., 2018, 2020), and (ii) were strongly correlated with clinical indices of disease severity (Child-Pugh score, MELD score, bilirubin) (Figure 3C and Supplementary Table 6). D-lactate was also positively correlated with total leukocyte, neutrophil and monocyte counts, and with all the cytokine measurements (Figure 3C and Supplementary Table 6). D-lactate and soluble-CD163 were also positively correlated to soluble-TIM3 and soluble-CD80 (Figure 3C and Supplementary Table 6) reinforcing the association between bacterial translocation, hyper-inflammation, soluble-CRs and disease severity in ALD patients.

### Investigating the Source of Soluble-TIM3 in ALD Patients and the Relationship Between Soluble-TIM3 and Membrane-TIM3 in Response to Bacterial Challenge

**FIGURE 4:**
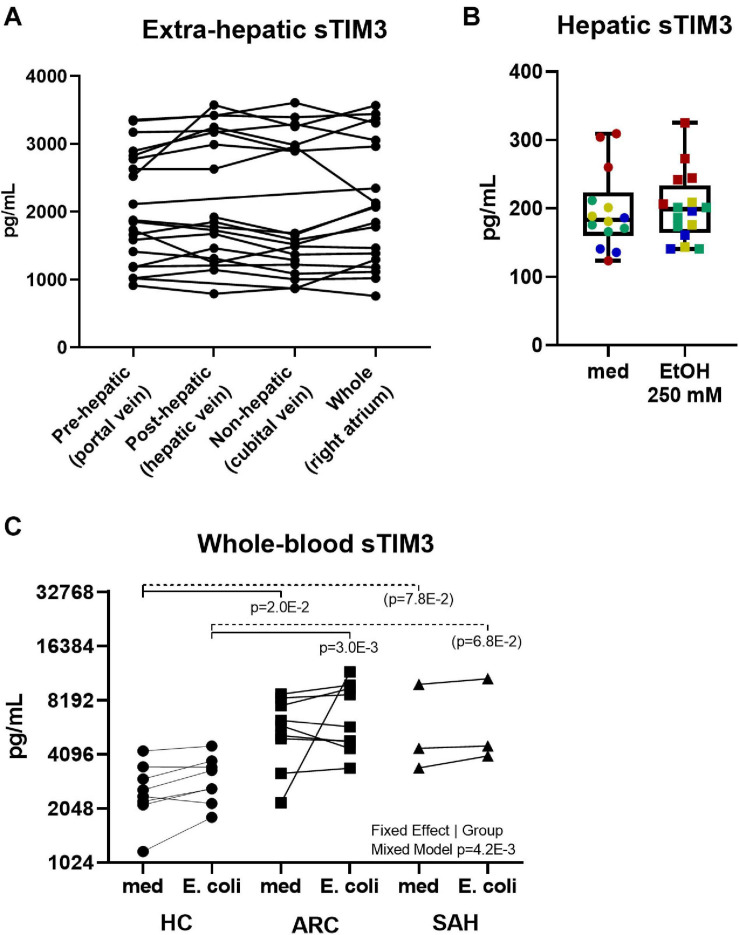
Anatomical distribution and peripheral origin of soluble-TIM3. **(A)** No differences in soluble-TIM3 measured in plasma obtained from four anatomical compartments in 20 ARC patients undergoing TIPS; measurements are matched by subject (Friedman’s paired test). **(B)** Lack of soluble-TIM3 production in PCLS treated with ethanol 250 mM for 24 h (Mann–Whitney test. Each dot represents one slice; each color represents one subject). Boxplots (median, IQR, ±full range). **(C)** Unchanged soluble-TIM3 levels in *E. coli*-pulsed whole blood (Mixed Model analysis, with significant fixed effect by subject group but not by stimulation).

To investigate the source of soluble-TIM3, soluble-CR concentrations were measured in plasma samples obtained from pre-hepatic (portal vein), post-hepatic (hepatic vein), and systemic (cubital vein and right cardiac atrium) blood beds in ARC patients during TIPS procedure. As shown in Figure 4A, intestinal/splanchnic or hepatic alterations during established ALD did not affect systemic levels of soluble-TIM3, and similar results were also obtained for all the soluble-CRs measured (Supplementary Figure 2).

To assess whether an acute alcohol ‘hit’ would trigger hepatic soluble-TIM3 production, human precision cut liver slices (PCLS) were treated with ethanol 250 mmol/L for 24 h but no release of soluble-TIM3 was observed upon this treatment, as shown in Figure 4B. This was also true for all the other soluble-CRs (Supplementary Figure 3A). Liver slices retained the immune infiltrate, viability (Supplementary Figure 3B) and tissue architecture during the culture period (Supplementary Figure 3C).

To investigate whether an acute bacterial challenge could provoke release of soluble-TIM3 in the whole blood, we measured soluble-CR concentrations in whole blood cultured with *E. coli* from ALD or HC subjects. As shown in Figure 4C, bacterial stimulation did not induce release of soluble-TIM3 or all the other soluble-CRs measured (Supplementary Figure 4). Of note, whole-blood soluble-TIM3 levels (in the absence of bacterial stimulation) were only different between groups (Figure 4C), in line with the plasma findings.

**FIGURE 5:**
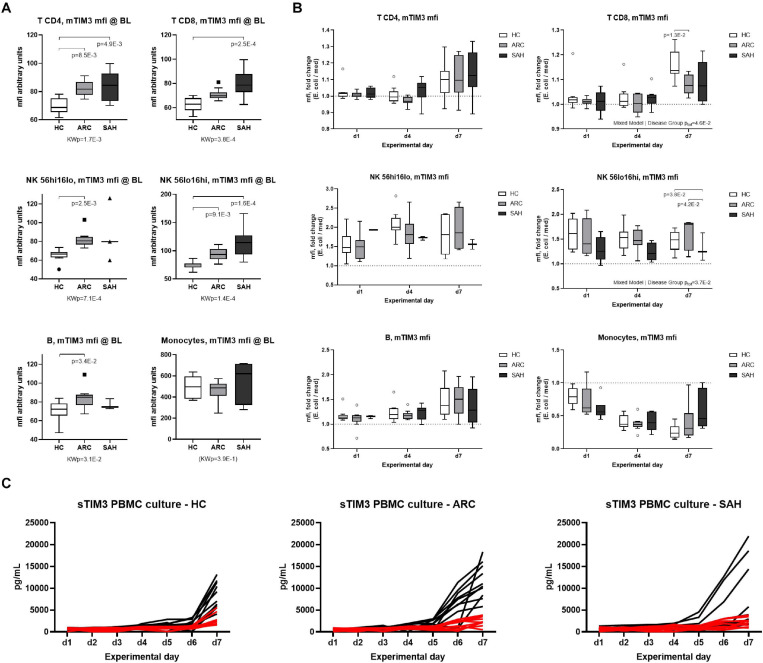
Expression of membrane-TIM3 on immune subsets and modulation by *E. coli*. **(A)** membrane-TIM3 mfi at baseline (day 1, unstimulated) on T-cells (CD4/CD8), NK-cells (CD56hi/CD56lo), B-cells and monocytes (Kruskal–Wallis test with Dunn’s multiple comparison correction). **(B)** Modulation of membrane-TIM3 mfi during a 7-day *E. coli*-stimulated culture; assessments performed at days 1–4–7; data expressed as *E. coli* fold ratio vs. unstimulated medium (Mixed Model analysis, with fixed effect comparisons by group). Boxplots: median, IQR, ±Tukey’s whiskers/outliers. **(C)** Soluble-TIM3 in daily PBMC culture supernatants; black lines indicate soluble-TIM3 levels in unstimulated medium; red lines represent soluble-TIM3 in *E. coli*-stimulated cultures. *N* = 10 for each HC, ARC, and SAH.

To assess whether soluble-TIM3 production from peripheral blood immune cells changes over time and to assess its relationship with membrane-TIM3 expression during anti-bacterial responses, membrane-TIM3 and soluble-TIM3 were measured daily by flow cytometry and Luminex assays, respectively, in *E. coli*-challenged PBMC cultures from ALD patients and HC (representative FACS gating shown in Supplementary Figure 5). Baseline unstimulated (basal) membrane-TIM3 expression was significantly higher in ALD compared to HC (SAH > ARC) on T, B, and NK-cells but not on monocytes (mfi, Figure 5A; %, Supplementary Figure 6A), confirming our previously published results (Markwick et al., 2015). Bacterial stimulation caused progressive changes of membrane-TIM3 expression. In particular, membrane-TIM3 increased on T, B, and NK-cells (mfi, Figure 5B; %, Supplementary Figure 6B) and decreased on monocytes (mfi, Figure 5B). These changes were comparable between ARC and HC but were less pronounced in SAH (Figure 5B and Supplementary Figure 6B). Measurements of soluble-TIM3 in cell-culture supernatants revealed lack of bacteria-stimulated soluble-TIM3 production by PBMCs in all groups (Figure 5C).

In correlation analyses (Supplementary Figure 7), plasma soluble-TIM3 was strongly positively correlated with basal membrane-TIM3% in CD4 T-cells and both NK-cell subsets (*r* ≥ 0.529, *p* ≤ 3.1E-2); with basal membrane-TIM3mfi in CD8 T-cells and both NK-cell subsets (*r* ≥ 0.529, *p* ≤ 1.8E-2); and with unstimulated membrane-TIM3mfi in day-4 CD56dim NK-cells and day-7 CD8 T-cells (*r* ≥ 0.474, *p* ≤ 4.0E-2). Regarding the changes of membrane-TIM3 expression induced by bacterial stimulation, plasma soluble-TIM3 was strongly negatively correlated with the early (day-1) membrane-TIM3 response in CD56dim NK-cells (% and mfi; *r* ≤ −0.475, *p* ≤ 4.0E-2); and with the membrane-TIM3 mfi response in both day-4 CD56dim NK-cells and day-7 CD8 T-cells (*r* ≤ −0.544, *p* ≤ 2.0E-2). Overall, subjects with high baseline plasma levels of soluble-TIM3 had higher basal membrane-TIM3 expression on NK-/T-cell subsets but also significantly weaker/null membrane-TIM3 upregulation on CD56dim NK-cells and CD8 T-cells upon bacterial challenge.

### Soluble-TIM3 Competitively Activates Anti-bacterial Immunity in ALD Patients

**FIGURE 6:**
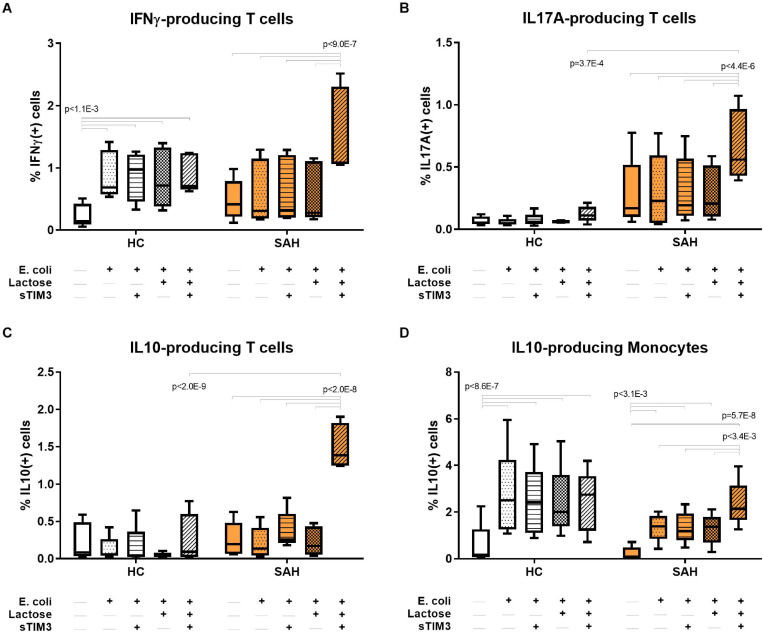
Soluble-TIM3 pathway and soluble-TIM3 functional characterisation. **(A)** Recombinant human soluble-TIM3 (rhsTIM3-Ig fusion protein) shows a stimulatory effect on *E. coli*-stimulated T-cell production of IFNγ, **(B)** T-cell IL-17A, **(C)** T-cell IL-10, and **(D)** monocyte IL-10 in PBMC cultures from SAH, after Galectin-9 binding neutralization with alpha-lactose; no effects observed in HC PBMC cultures (2-way ANOVA with Holm–Sidak’s *post hoc* correction for paired samples); orange bars represent SAH PBMCs; white bars represent HC PBMCs; shading identifies different culture conditions as specified along each graph’s *x*-axis. Boxplots: median, IQR, ±Tukey’s whiskers/outliers.

To investigate the biological function of soluble-TIM3 in ALD patients, in the context of anti-bacterial responses, the TIM3 receptor-ligand equilibrium was assessed with *in vitro* PBMC cultures by neutralizing Galectin-9 with alpha-lactose and by competitive saturation using an excess of recombinant human soluble-TIM3-Ig fusion protein (rhsTIM3-Ig). As illustrated in Figure 6A, PBMC stimulation with *E. coli* induced T-cell IFNγ only in HC but not SAH, confirming our previously published observations (Markwick et al., 2015; Riva et al., 2018). T-cell production of anti-bacterial IL-17A and anti-inflammatory IL-10 were unchanged (Figures 6B,C) and monocyte IL-10 was induced in both groups (Figure 6D). Pre-treatment with rhsTIM3-Ig alone had no effect on *E. coli*-stimulated cytokines (Figures 6A–D). However, following Galectin-9 neutralization with alpha-lactose, rhsTIM3-Ig unlocked anti-bacterial T-cell IFNγ (Figure 6A) and IL-17A (Figure 6B) production in SAH patients, and strongly stimulated their T-cell/monocyte IL-10 production (Figures 6C,D, respectively). Of note, this immune reactivation was selective for ALD patients (SAH), as neither treatments elicited any effects in HC.

Control experiments on Galectin-9 neutralization in PBMCs from SAH patients stimulated with *E. coli* in the presence or absence of a blocking anti-Galectin-9 monoclonal antibody (Supplementary Figure 8) indicated that Galectin-9 blockade alone was not sufficient to recapitulate all the changes observed when Galectin-9 neutralization was achieved with alpha-lactose and excess rhsTIM3-Ig. In particular, Galectin-9 blockade could not rescue the production of *E. coli*-stimulated anti-bacterial IFNγ (Supplementary Figure 8A), while its effects on the production of pro-inflammatory IL-17A by CD8 T-cells (Supplementary Figure 8B) and anti-inflammatory IL-10 by monocytes (Supplementary Figure 8D) were only marginal.

## Discussion

We have previously shown that the compromised innate and adaptive anti-bacterial immunity in SAH could be maneuvered to a more favorable state through *ex vivo* neutralization of membrane-bound checkpoint receptors TIM3 and PD1 on lymphocytes, without inducing the production of cytokines associated with systemic inflammatory response syndrome (Markwick et al., 2015). However, the role of the recently discovered functional soluble (cell-free) CRs in the immune dysregulation associated with ALD is not well understood and this was the aim of the current study.

The main finding from our analysis is that soluble-TIM3 and its soluble ligands Galectin-9 and soluble-CEACAM1 are closely associated with the severity of ALD. Multivariate assessments (PCA and PLS-DA) of soluble-CRs highlighted disease-based differences among subjects and indicated soluble-TIM3 as the strongest driver of these differences in ALD. Soluble-TIM3 was also strongly correlated with a highly inflammatory state, increased bacterial translocation and clinical indices of disease severity in ALD patients, highlighting a novel potential immune-related pathogenetic mechanism associated with disease progression. These findings led us to focus our subsequent work on the characterisation of the soluble-TIM3 pathway and its role in ALD.

The immunosuppressive activity of membrane-TIM3 has been characterized in detail; the engagement of membrane-TIM3 by its ligands Galectin-9 and CEACAM1 activates downstream intracellular signaling pathways leading to functional inactivation of the immune cell (Davidson et al., 2007; Lee et al., 2011; Rangachari et al., 2012; Clayton et al., 2014; Das et al., 2017). Blocking membrane-TIM3 with neutralizing antibodies prevents T-cell ‘shutdown’ and reactivates immune function (Sabatos-Peyton et al., 2018), underpinning pre-clinical and clinical studies for TIM3-based immunotherapy (Riva and Chokshi, 2018; Friedlaender et al., 2019). In the present study, we found that during ALD, and predominantly in SAH patients, membrane-TIM3 is hyper-expressed on immune cells, reconfirming previously published results (Markwick et al., 2015), and plasma levels of Galectin-9 and CEACAM1 are also highly increased. In these circumstances, over-expression of both ligands and their membrane receptor may drive widespread immune impairment. However, the soluble cell-free form of TIM3 is known to act as a ‘decoy antagonist’ and when plasma levels are supra-physiological, like those found in ALD patients in the present study, sequestration of Galectin-9 and CEACAM1 may prevent immune suppression mediated via membrane-TIM3 (Jones et al., 2008; Muthukumarana et al., 2008; Vega-Carrascal et al., 2011; Nebbia et al., 2012; Wu et al., 2012; de Kivit et al., 2013, 2017; Xiao et al., 2013; Kojima et al., 2014). In fact, detectable complexes of soluble-TIM3 and Galectin-9 have been described in the literature (Silva et al., 2017) and we would suggest that the heightened production of soluble-TIM3 in ALD could be an attempt to counter the immunosuppressive properties of Galectin-9 and CEACAM1.

However, the binding between TIM3, Galectin-9 and CEACAM1 does not occur in a 1-to-1 ratio. Whilst TIM3 is monomeric, Galectin-9 is homodimeric and both Galectin-9 and CEACAM1 are molecular mixtures of several isoforms (Zhu et al., 2005; Nagaishi et al., 2008; Huang et al., 2015; Horst et al., 2018; Kim et al., 2019). Thus, even when quantitative molar ratios are comparable, the number of binding sites for TIM3 may be in excess. As such, Galectin-9 and CEACAM1 could act as ‘molecular sponges’ for soluble-TIM3 while still retaining enough unsaturated binding sites to ligate membrane-TIM3 on immune cells, thereby suppressing immune responses and inhibiting anti-bacterial immunity.

To explore this, we saturated bacteria-stimulated PBMC cultures with recombinant human soluble-TIM3 (rhsTIM3-Ig fusion protein) and biochemically neutralized Galectin-9 with alpha-lactose; we found that this cocktail was able to unlock anti-bacterial immunity (IFNγ and IL-17) and restore anti-inflammatory IL-10 production in AH patients. As expected, immune rescue was not observed in controls, where the TIM3 pathway was not dysregulated. Notably, Galectin-9 blockade alone with a neutralizing anti-Gal9 monoclonal antibody was not sufficient to recapitulate the immune rescue effects observed when soluble-TIM3 was used in combination with alpha-lactose. It is important to mention that several publications confirm that the type of recombinant human soluble-TIM3 used in the present study can selectively bind TIM3 ligands, is biologically active and has comparable blocking activity to anti-TIM3 antibodies (Vega-Carrascal et al., 2011; Nebbia et al., 2012; Wu et al., 2012; de Kivit et al., 2013, 2017; Kojima et al., 2014). The *in vitro* immune rescue activity of this rhsTIM3-Ig has been shown to be highly antigen-specific, with no effects upon generalized stimuli such as PMA/Ionomycin or anti-CD3/CD28 (Nebbia et al., 2012; Wu et al., 2012), which may be relevant in the context of bacteria-specific responses. Furthermore, rhsTIM3-Ig has also demonstrated the ability to enhance compensatory type-2 responses (Wang et al., 2015) potentially dampening type 1 inflammation, which may be highly relevant in ALD and specifically SAH patients where chronic inflammation underlies immunopathology. Taken together our study would suggest that TIM3 ligands Galectin-9 and soluble-CEACAM1 may be central to the immune impairment in AH and that therapeutic administration of soluble-TIM3 may be a novel option to improve anti-bacterial effector functions in AH.

We next turned our attention to investigate the possible sources of soluble-TIM3 in ALD. Firstly we aimed to understand if there was a direct and temporal relationship between the expression of membrane-TIM3 on immune cells and the production of the soluble form, and whether the latter was upregulated during bacterial challenge. During *E. coli* challenge of PBMCs, we observed progressive loss of membrane-TIM3 expression in monocytes from both ALD patients and controls, and dampened membrane-TIM3 upregulation on Natural Killer cells and CD8 T-cells from ALD patients, the latter being in strong negative correlation with higher plasma soluble-TIM3 in the same patients. However, these membrane-TIM3 changes were not accompanied by increased soluble-TIM3 in our culture supernatants upon *E. coli* stimulation. Notably, it has been shown that monocytes (Moller-Hackbarth et al., 2013) and activated CD8 T-cells (Clayton et al., 2015) release soluble-TIM3 in response to lipopolysaccharide and other broad stimulators such as PMA/Ionomycin, but no information is available on membrane-TIM3 and soluble-TIM3 kinetics during direct bacterial stimulation. More research is needed to clarify whether direct bacterial stimulation acts differently on soluble-TIM3 shedding or other mechanisms are at play, such as recapture, proteolytic degradation, sequestration or epitope masking.

Next, we assessed whether systemic soluble-TIM3 originated from the liver during ALD. Using a novel human immunocompetent precision cut liver slice model of ALD, we found that upon treatment with high ethanol concentrations, soluble-TIM3 levels were not prompted. This finding was confirmed in plasma from patients undergoing TIPS procedure, where soluble-TIM3 concentrations were measured in plasma samples obtained from pre-hepatic, post-hepatic, and systemic blood beds in ARC patients: soluble-TIM3 levels were high but not different between these compartments.

Rather than changes in a specific source, the elevated soluble-TIM3 levels may be due to activity of metalloproteases involved in soluble-TIM3 shedding, such as ADAM10 or ADAM17, which warrants further investigation. In fact, it is well described that inflammatory responses lead to increased metalloprotease activity and in these conditions production of soluble-TIM3 by proteolytic cleavage may be facilitated (Moller-Hackbarth et al., 2013; Clayton et al., 2015; Ren et al., 2015). However, their ubiquitous expression, relative lack of specificity and broadly diverse biological roles *in vivo* (Weber and Saftig, 2012; Giebeler and Zigrino, 2016; Wetzel et al., 2017; Zunke and Rose-John, 2017; Saha et al., 2019) would make them difficult therapeutic targets.

Interestingly, the soluble form of PD1 and its ligands were not different between groups, despite our previous finding of membrane-PD1 being upregulated on immune cells in ALD patients (Markwick et al., 2015). There are several soluble isoforms of PD1, one of which retains ligand binding capacity and is therefore putatively biologically active (Nielsen et al., 2005), but whether our quantification measured total soluble-PD1 or any of its isoforms is unclear, as currently no commercially available reagents allow the measurement of distinct isoforms. The existence of different soluble isoforms may affect other soluble-CRs as well, including soluble-CD80 (Lahat et al., 2003; Kakoulidou et al., 2007), soluble-LAG3 (Li et al., 2007), soluble-HVEM (Heo et al., 2012; Li et al., 2020), and the already discussed soluble-TIM3 (Moller-Hackbarth et al., 2013; Clayton et al., 2015; Ren et al., 2015), Galectin-9 and CEACAM1 (Zhu et al., 2005; Nagaishi et al., 2008; Huang et al., 2015; Horst et al., 2018; Kim et al., 2019), and we believe this also to be the reason for the discrepancy between our findings and a recent publication by Li et al. (2020) where soluble-HVEM was found to be the most significantly dysregulated soluble-CR in AH patients (Wang et al., 2001; Heo et al., 2012; Zhao Q. et al., 2017; Li et al., 2020). There is currently no information on the relative balance between soluble-CR isoforms in health and disease, but since changes in this equilibrium may have pathophysiological consequences, this may be important to discern for future advances in the field.

To summarize, the data from this study reveal new insights in the immunopathogenesis of ALD. In particular, we describe a novel and highly dysregulated soluble-TIM3/ligand axis in ALD, which may offer new immunomodulatory approaches to restore an effective state of anti-pathogen host defense in these highly immunocompromised patients.

## Lay Summary

Alcohol-related liver disease (ALD) is an escalating global problem and is responsible for >2.5 million deaths/year. One of the major and most common complications that ALD patients face is an increased susceptibility to infection, which can lead to worsening of liver disease and multi-organ failure. In fact, the development of bacterial infection often signals the terminal phase in ALD, increasing the probability of death to 30% at 1 month and >60% by 1 year. The mechanisms responsible for this susceptibility are not well understood and it is vital that we understand why patients with alcohol-related liver injury have impaired defenses against infection and the factors that may influence this, in order to develop new treatments beyond the use of antibiotics, which are driving the selection of multi-drug resistant bacteria in this patient group. In this study, we present novel findings describing the central role of the immunoregulatory molecule TIM3 and its binding partners Galectin-9 and CEACAM1 in the impaired immunity in ALD patients. We also show that the soluble form of TIM3 may be a promising therapeutic target to facilitate rescue of anti-microbial immunity without exacerbating damaging inflammatory responses.

## Data Availability Statement

The data supporting the conclusions of this article may be made available by the authors, without undue reservation, to any qualified researcher upon reasonably justified request.

## Ethics Statement

The studies involving human participants were reviewed and approved by United Kingdom Research Ethics Committee reference numbers 13/SW/0219, 08/H0702/52, 12/SC/0359, and 17/NE/0340; Bulgarian Ethics Protocol 1/27.02.18; and Bonn University Ethics Committee reference number 029/13. The patients/participants provided their written informed consent to participate in this study.

## Author Contributions

AR: study design and supervision, experimental work, data acquisition, analysis, presentation, manuscript preparation, and revision. SC: study design and supervision, funding, manuscript revision, and final approval. EP, DD, and DC: additional laboratory work. DC, HA, NH, KM, MeP, AZ, RM, JR, GW, SF, AE, DS, RS, SK, FU, MiP, KK, TH, SP, MS, and JT: provision of samples. RW: critical evaluation of the manuscript for important intellectual content and study funding. All authors reviewed and approved the final version of the manuscript.

## Conflict of Interest

The authors declare that the research was conducted in the absence of any commercial or financial relationships that could be construed as a potential conflict of interest.

## Abbreviations

ALD: alcohol-related liver disease
ARC: alcohol-related cirrhosis
BH FDR: Benjamini–Hochberg False Discovery Rate
BHq: BH FDR adjusted *p*-value (i.e. *q*-value)
BTLA: B- and T-lymphocyte attenuator
C.I.: confidence interval
CD: cluster of differentiation
CEACAM1: carcinoembryonic antigen-related cell adhesion molecule 1
DMSO: dimethyl sulfoxide
Gal9: galectin-9
GITR: glucocorticoid-induced TNFR-related protein
HC: healthy controls
HVEM: herpesvirus entry mediator
IDO: indoleamine 2,3-dioxygenase
IFN: interferon
IL: interleukin
IQR: interquartile range
KWp: Kruskal–Wallis *p*-value
LAG3: lymphocyte-activation gene 3
MELD: model end-stage liver disease
membrane-CR(s): membrane checkpoint receptor(s)
mfi: median fluorescence intensity
MWp: Mann–Whitney *p*-value
PBMC(s): peripheral blood mononuclear cell(s)
PBS: phosphate-buffered saline
PD1: programmed death 1
PDL1/PDL2: programmed death ligand 1/2
rcf: relative centrifugal force
(S)AH: (severe) alcoholic hepatitis
soluble-CR(s): soluble checkpoint receptor(s)
TIM3, T-cell immunoglobulin and mucin domain 3 (also known as Hepatitis A virus cellular receptor 2: HAVCR2)
TNF: tumor necrosis factor
