# Supplementary material for: Soluble TIM3 and Its Ligands Galectin-9 and CEACAM1 Are in Disequilibrium During Alcohol-Related Liver Disease and Promote Impairment of Anti-bacterial Immunity
Source: Front Physiol. 2021 Mar 10;12:632502. doi: 10.3389/fphys.2021.632502 (PMC7987668; doi:10.3389/fphys.2021.632502)
Supplement: Supplementary file 1 [file Data_Sheet_1.PDF]

Soluble TIM3 and its ligands Galectin-9 and CEACAM1 are in disequilibrium during Alcohol-related Liver Disease and promote impairment of anti-bacterial immunity.

Antonio Riva[1,2,*], Elena Palma[1,2], Dhruti Devshi[1,2], Douglas Corrigall[1,2,3], Huyen Adams[1,2,4], Nigel Heaton[5], Krishna Menon[5], Melissa Preziosi[5], Ane Zamalloa[5], Rosa Miquel[6], Jennifer M Ryan[7], Gavin Wright[3], Sarah Fairclough[3], Alexander Evans[4], Debbie Shawcross[2], Robert Schierwagen[8], Sabine Klein[8], Frank E Uschner[8], Michael Praktiknjo[9], Krum Katzarov[10], Tanya Hadzhiolova[10], Slava Pavlova[10], Marieta Simonova[10], Jonel Trebicka[8,11], Roger Williams[1,2], Shilpa Chokshi[1,2,*]

1 Institute of Hepatology (Foundation for Liver Research), London, UK
2 Faculty of Life Sciences and Medicine (King's College London), London, UK
3 Department of Gastroenterology, Basildon University Hospital, Basildon, UK
4 Department of Gastroenterology, Royal Berkshire Hospital, Reading, UK
5 Institute of Liver Studies (King's College London), London, UK
6 Liver Histopathology Laboratory, Institute of Liver Studies, King's College Hospital
7 Gastrointestinal and Liver Services, Royal Free Hospital, London, UK
8 Translational Hepatology, Department of Internal Medicine I, University Clinic Frankfurt, Frankfurt, Germany
9 Department of Internal Medicine I, University of Bonn, Bonn, Germany
10 Department of Gastroenterology, Hepatobiliary surgery and Transplantology, Military Medical Academy, Sofia, Bulgaria
11 European Foundation for the Study of Chronic Liver Failure, EF-CLIF, Barcelona, Spain
* Corresponding authors

Short title: Soluble immune checkpoints in ALD
Keywords: TIM3; immune checkpoint; alcohol; biomarker

Supplementary Figures and Tables

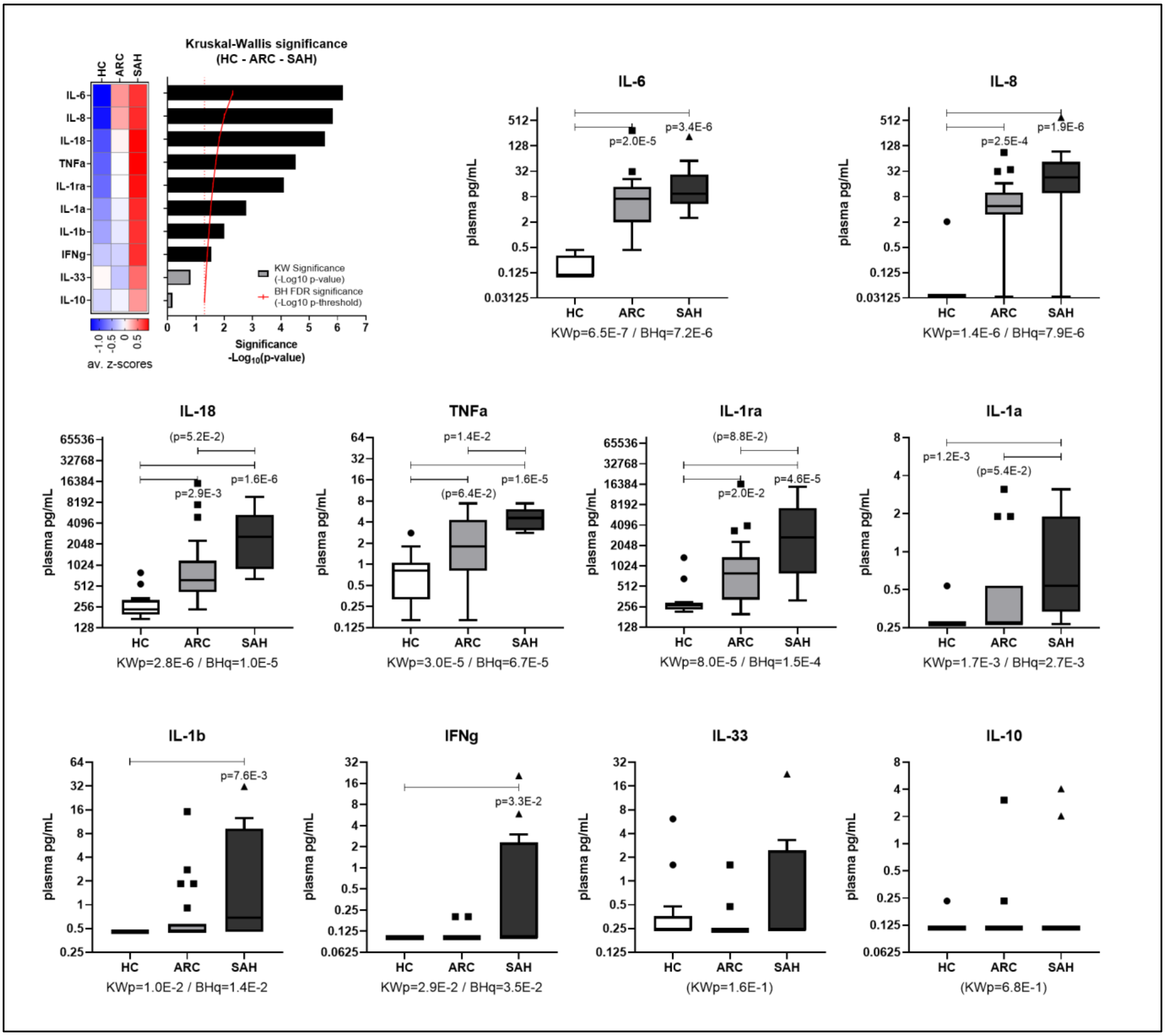

Supplementary figure 1

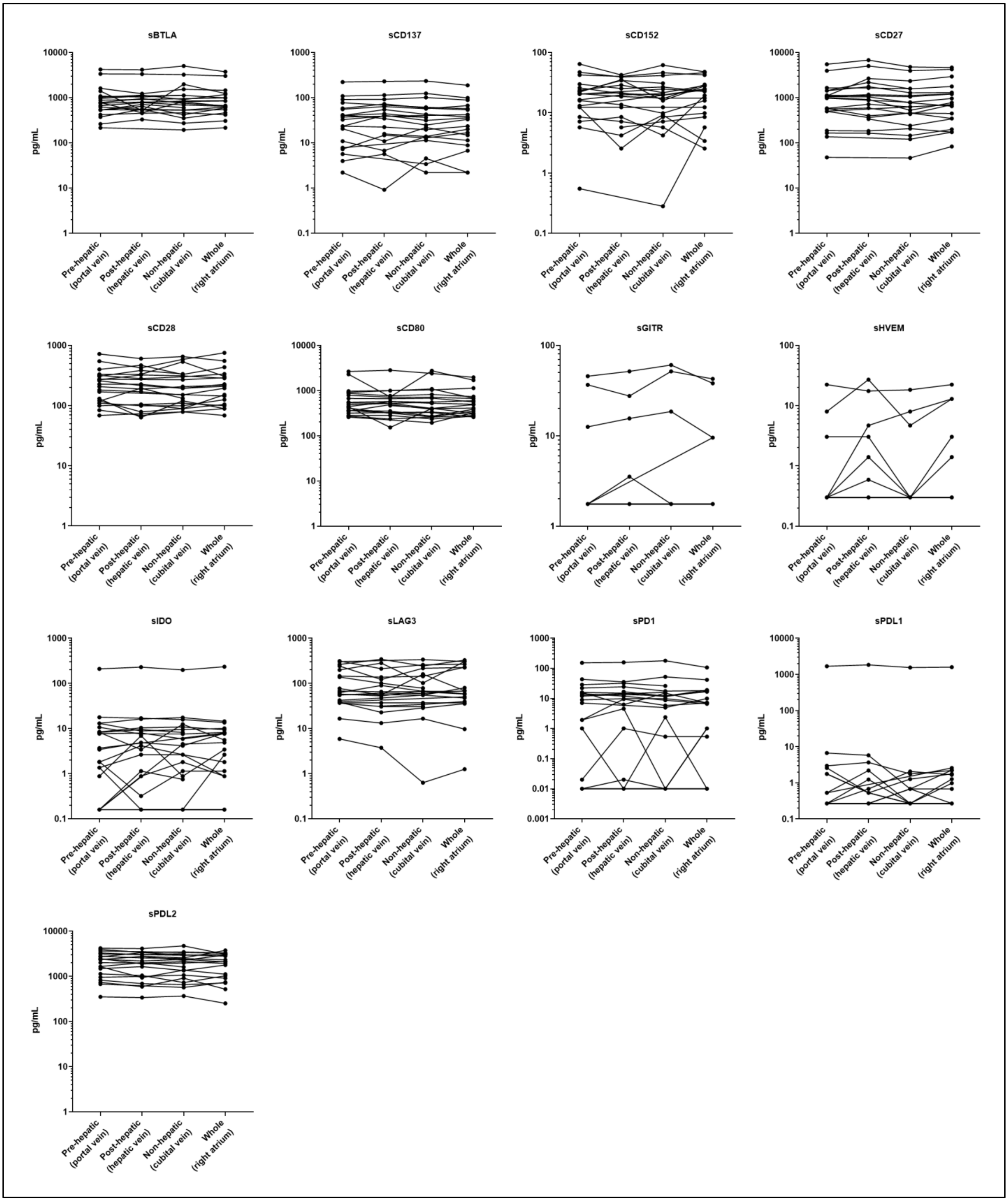

Supplementary figure 2

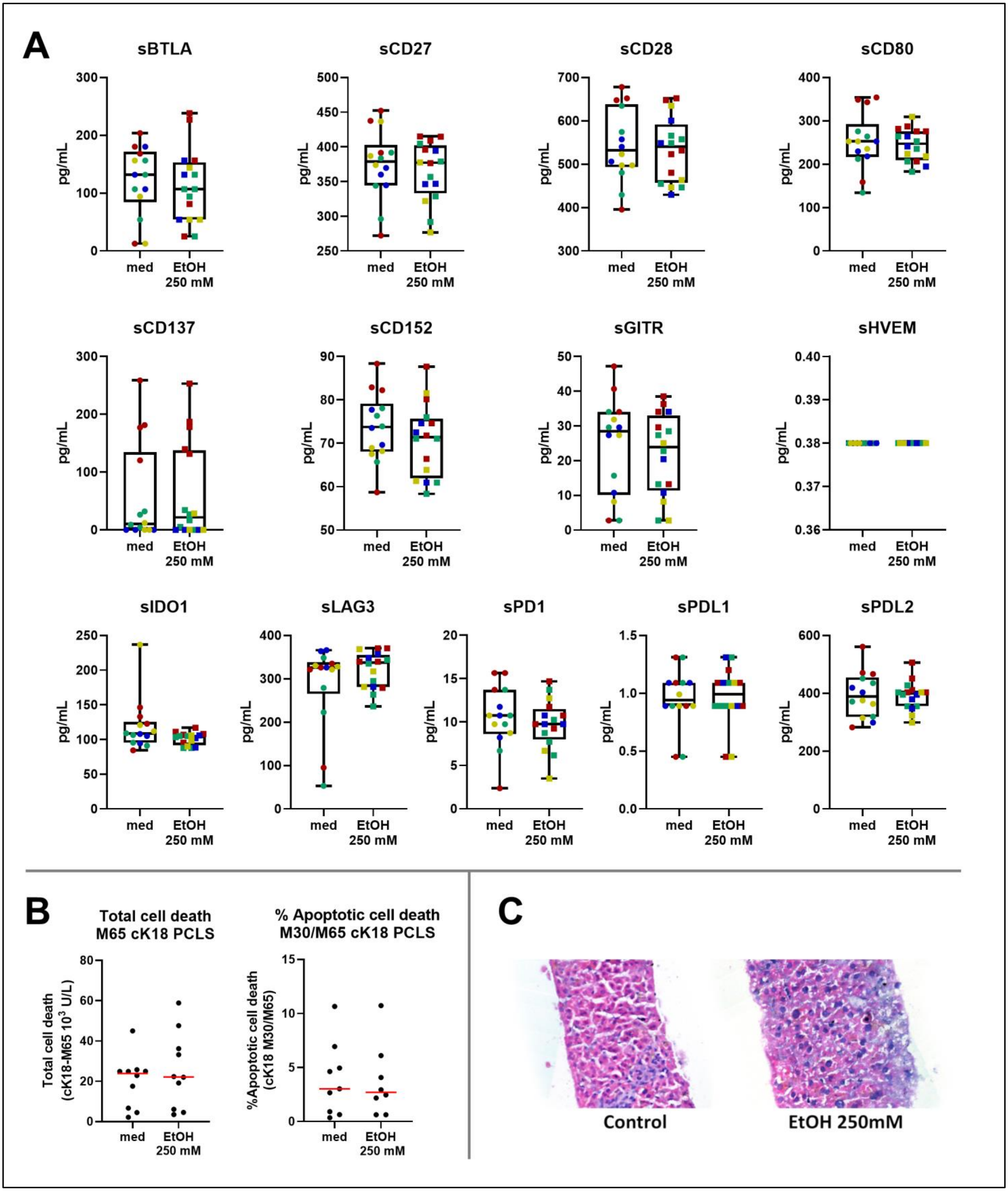

Supplementary figure 3

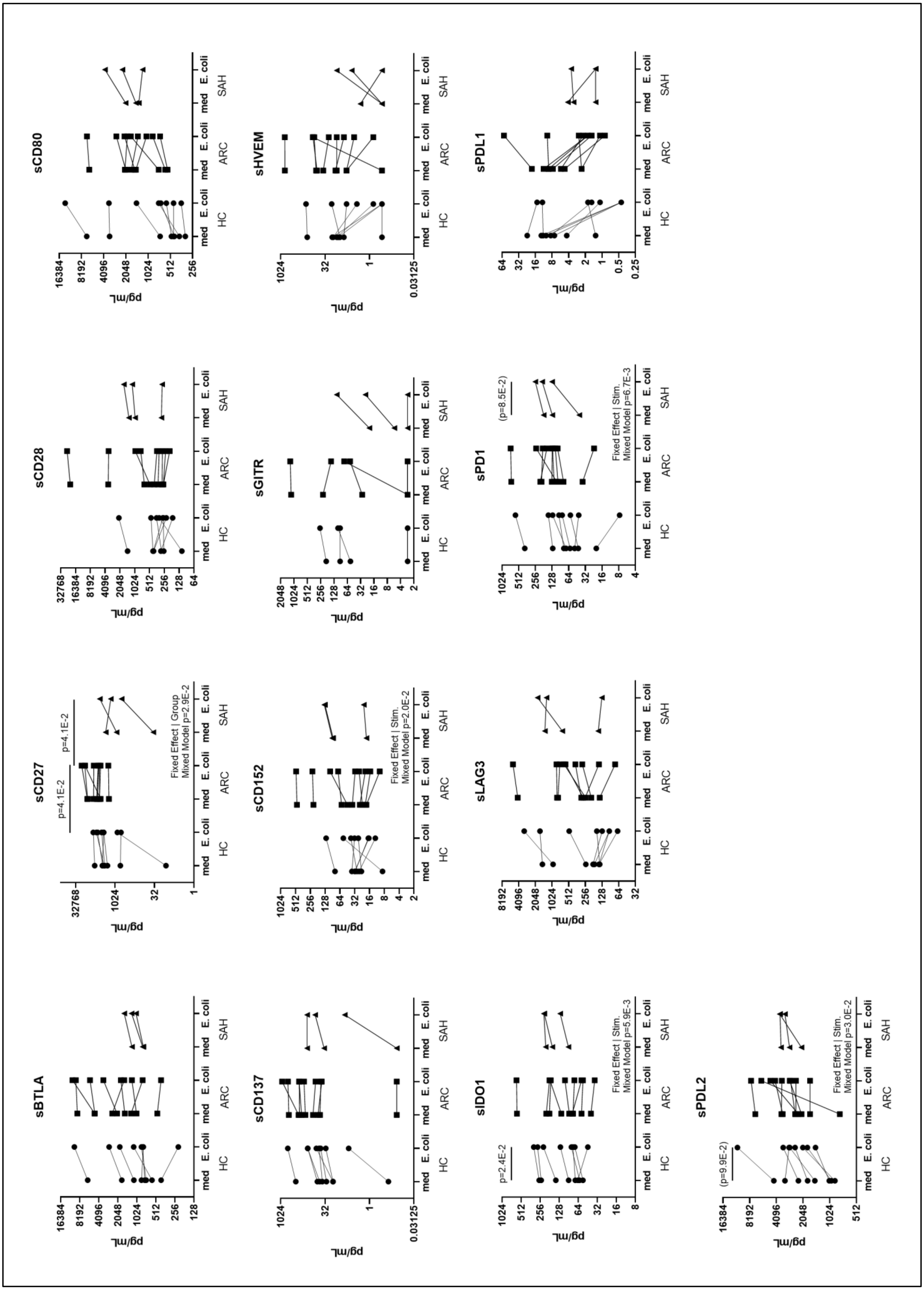

Supplementary figure 4

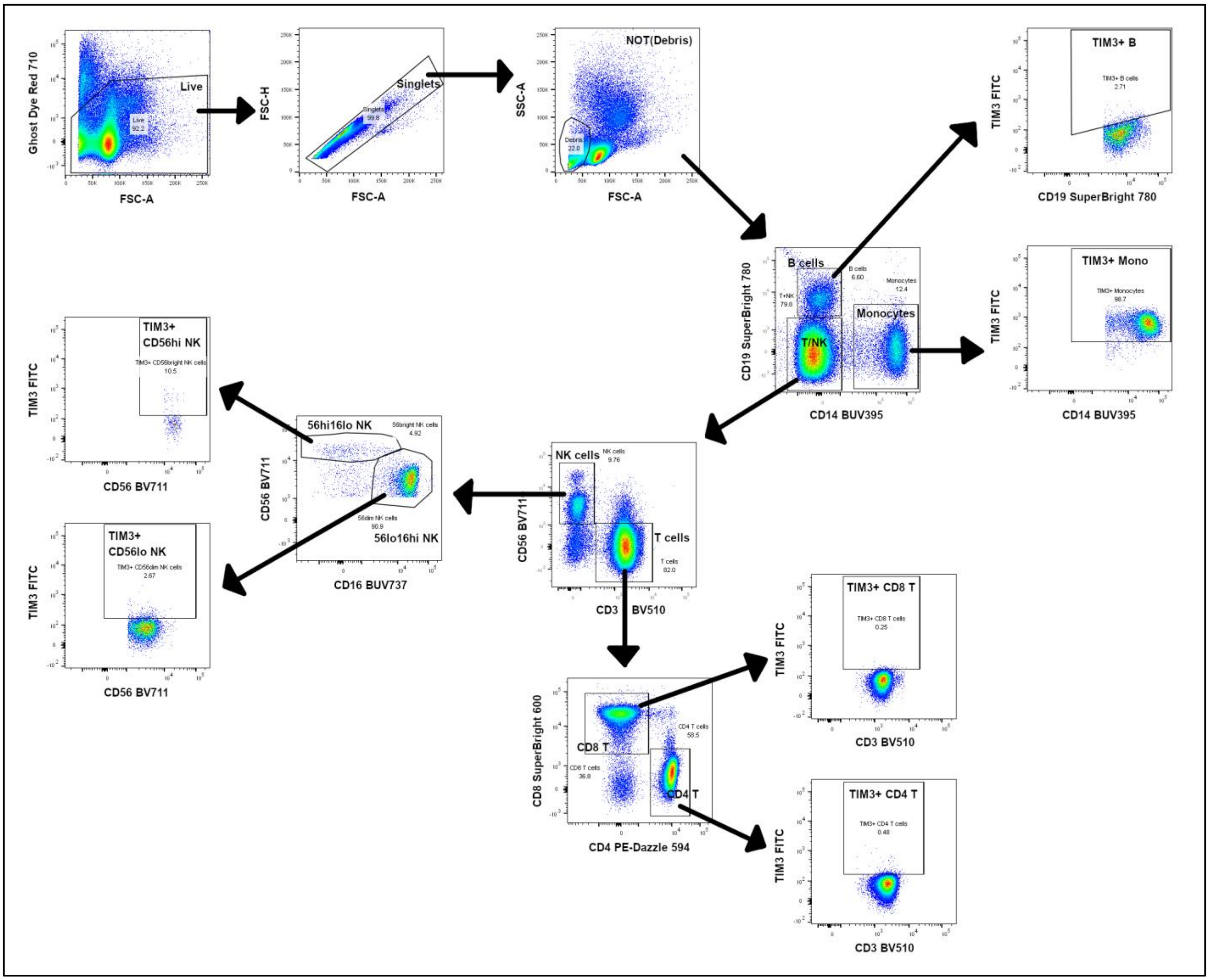

Supplementary figure 5

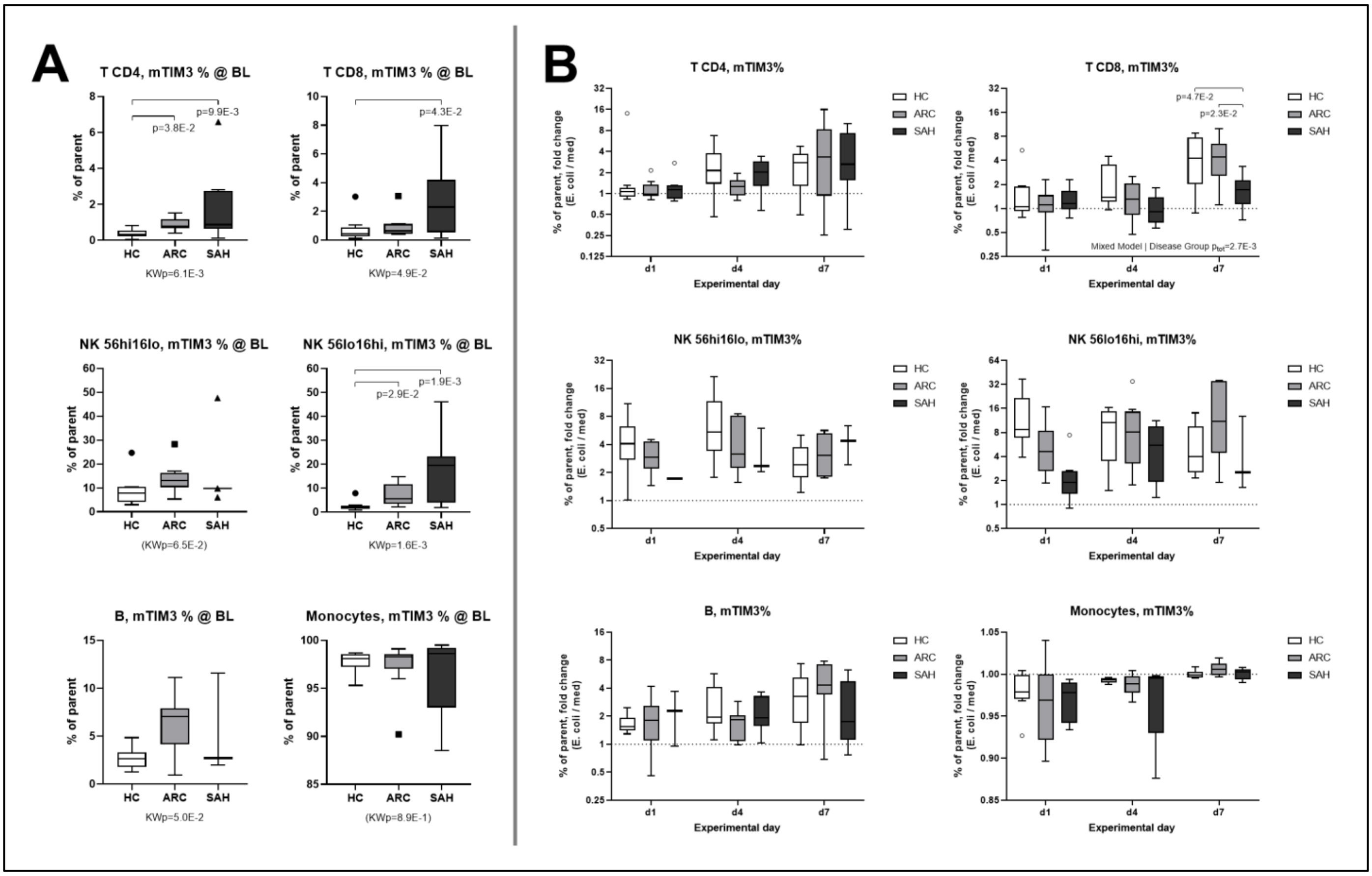

Supplementary figure 6

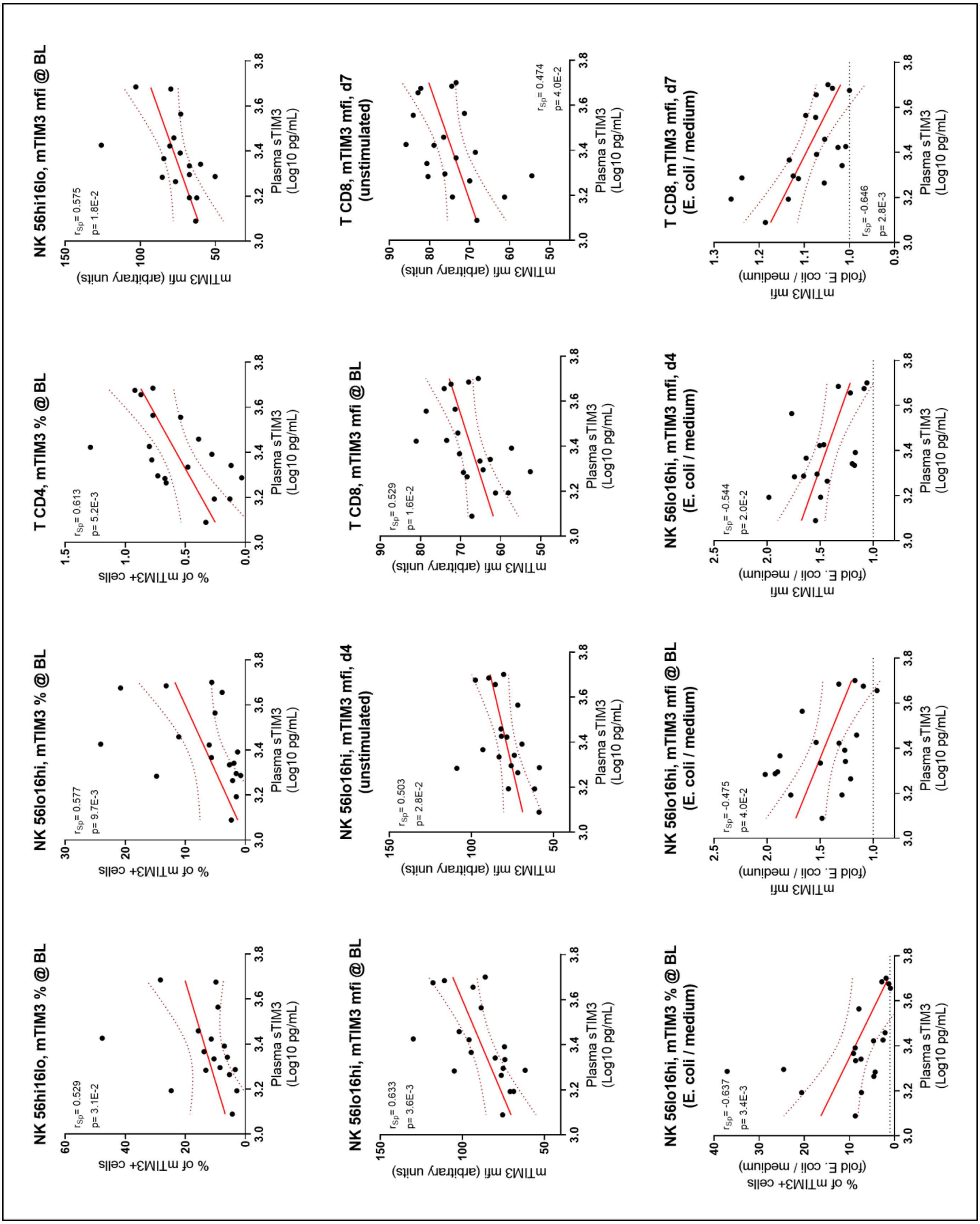

Supplementary figure 7

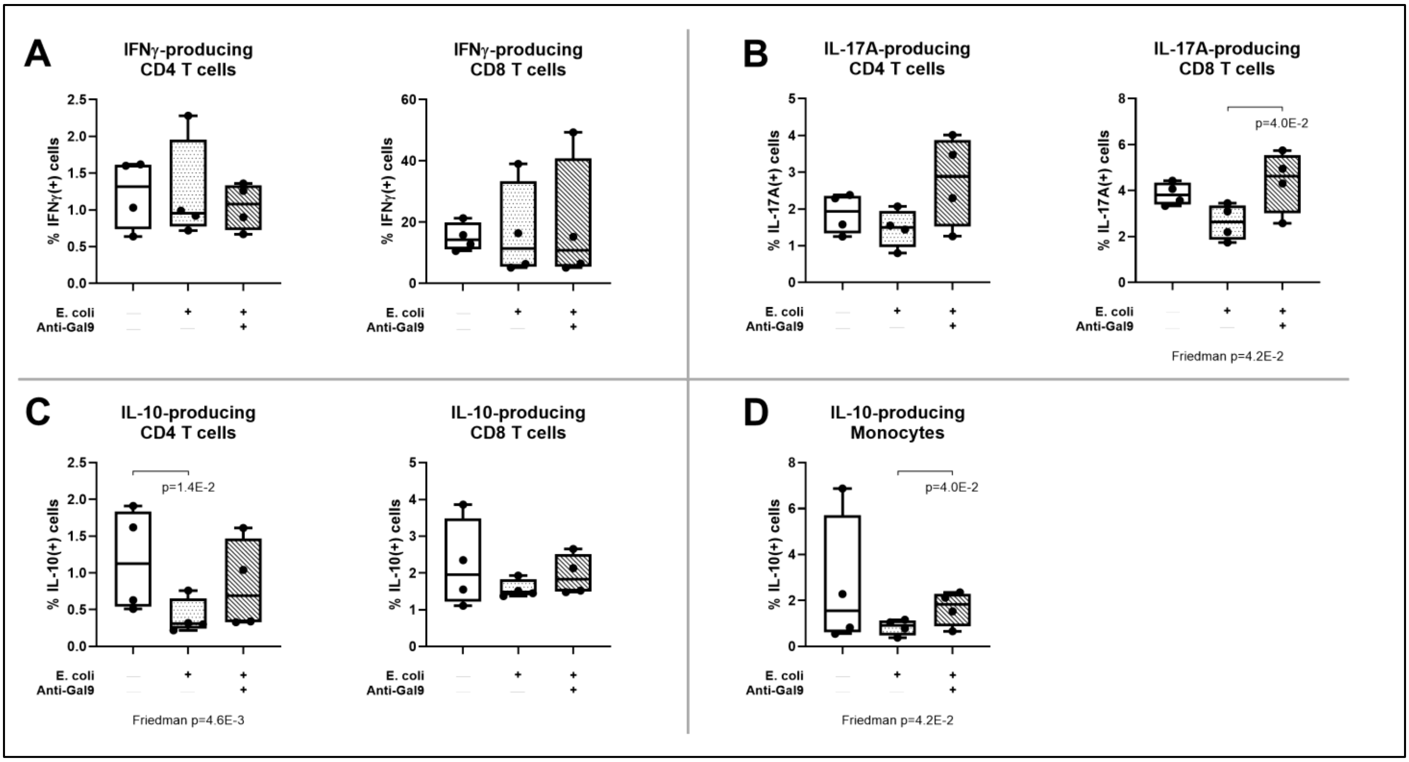

Supplementary figure 8

FACS panel for mTIM3 assessment

Marker	Color/Format	Clone	Laser/Ex Filter		Em Filter	Company	Catalog
**CD14**	BUV395	MphiP9	UV	355	379/28	BD Biosciences	563561
**CD16**	BUV737	3G8	UV	355	740/35	BD Biosciences	612786
**CD3**	Brilliant Violet 510	UCHT1	V	405	525/50	BioLegend	300448
**CD8a**	SuperBright 600	SK1	V	405	610/20	Thermo Fisher Scientific	63-0087-42
**CD56**	Brilliant Violet 711	HCD56	V	405	710/50	BioLegend	318336
**CD19**	SuperBright 780	SJ25C1	V	405	780/60	Thermo Fisher Scientific	78-0198-42
**TIM3**	FITC	F38-2E2	B	488	530/30	Thermo Fisher Scientific	11-3109-42
**CD4**	PE-Dazzle 594	OKT4	YG	561	610/20	BioLegend	317448
**Ghost Dye Red 710**	Ghost Dye Red 710	N/A	R	640	730/45	Tonbo Biosciences (Cambridge Bioscience)	13-0871-T500

FACS panel for rhsTIM3-Ig cultures

Marker	Color/Format	Clone	Laser/Ex Filter		Em Filter	Company	Catalog
**CD3**	Brilliant Violet 510	UCHT1	V	405	525/50	BioLegend	300448
**IFNg**	Alexa Fluor 488	4S.B3	B	488	530/30	BioLegend	502515
**IL-10**	PE	JES3-9D7	B	488	585/42	BioLegend	501404
**CD8a**	PerCP-eFluor710	SK1	B	488	670 LP	eBioscience	46-0087-42
**IL-17**	Alexa Fluor 647	SCPL1362	R	633	660/20	BD	560437
**CD4**	APC-eFluor780	OKT4	R	633	780/60	eBioscience	47-0048-42

FACS panel for anti-Galectin-9 antibody cultures

Marker	Color/Format	Clone	Laser/Ex Filter		Em Filter	Company	Catalog
**CD14**	BUV395	MphiP9	UV	355	379/28	BD Biosciences	563561
**CD3**	Brilliant Violet 510	UCHT1	V	405	525/50	BioLegend	300448
**CD8a**	SuperBright 600	SK1	V	405	610/20	Thermo Fisher Scientific	63-0087-42
**IL-17**	Brilliant Violet 650	BL168	V	405	670/30	BioLegend	512304
**IL-10**	FITC	4S.B3	B	488	530/30	Thermo Fisher Scientific	BMS131-2FI
**IFNg**	PE	25723	YG	561	586/16	R&D	IC285P-100
**CD4**	PE-Dazzle 594	OKT4	YG	561	610/20	BioLegend	317448
**Ghost Dye Red 710**	Ghost Dye Red 710	N/A	R	640	730/45	Tonbo Biosciences (Cambridge Bioscience)	13-0871-T500

Supplementary table 1

	Stats	HC	ARC	SAH
**sTIM3**	#5	2057.57 (1663.47 – 2179.89)	3912.93 (2756.22 – 4730.43)	4522.30 (3589.62 – 4726.76)
**sCD80**	#4	441.21 (337.02 – 470.16)	1172.48 (649.75 – 1938.85)	1493.36 (1009.56 – 2422.43)
**sLAG3**	#1	51.67 (46.64 – 76.94)	124.67 (66.15 – 196.88)	90.58 (73.67 – 107.46)
**sHVEM**	#1	141.37 (101.72 – 388.40)	82.18 (56.78 – 141.83)	52.45 (38.73 – 87.48)
**sPDL1**	(#1)	4.59 (3.96 – 10.22)	10.74 (8.59 – 16.58)	10.22 (7.17 – 11.27)
**sCD137**		89.18 (60.29 – 121.59)	130.65 (95.05 – 204.27)	126.13 (93.88 – 153.04)
**sCD27**		1874.70 (1504.41 – 2362.32)	2764.04 (1468.85 – 4952.98)	2774.13 (2631.39 – 4898.27)
**sPDL2**		1642.96 (1231.25 – 1829.19)	2217.21 (1534.49 – 3303.72)	2800.48 (1857.30 – 3321.46)
**sIDO**		24.43 (20.84 – 43.22)	34.89 (30.63 – 68.67)	38.26 (26.65 – 47.29)
**sGITR**		13.68 (7.93 – 77.36)	83.18 (44.02 – 145.72)	36.78 (25.22 – 158.82)
**sBTLA**		1202.58 (980.29 – 1583.70)	1491.51 (1034.11 – 2129.54)	1118.88 (952.71 – 1555.23)
**sCD28**		144.26 (126.08 – 233.04)	212.55 (144.26 – 322.07)	156.28 (138.23 – 197.86)
**sCD152**		44.23 (37.94 – 62.00)	51.36 (43.33 – 65.31)	45.12 (38.84 – 55.80)
**sPD1**		59.13 (41.16 – 108.18)	89.11 (47.57 – 163.74)	66.00 (48.52 – 105.04)

All measurements are in pg/mL. Data expressed as median (IQR).
# = significant difference between HC/ARC/SAH (Benjamini-Hochberg (BH) adjusted Kruskal-Wallis test)
1 symbol: BHq≤0.05 / 2 symbols: BHq≤0.01 / 3 symbols: BHq≤0.005 / 4 symbols: BHq≤0.001 / 5 symbols: BHq≤0.0005
(symbol) = non-significant trend, (p≤0.05) but BH-adjusted q>0.05

Supplementary table 2

Correlations														
	Log10_sBTLA	Log10_sCD137	Log10_sCD152	Log10_sCD27	Log10_sCD28	Log10_sCD80	Log10_sGITR	Log10_sHVEM	Log10_sIDO	Log10_sLAG3	Log10_sPD1	Log10_sPDL1	Log10_sPDL2	Log10_sTIM3
p-value	Sig. (2-tailed)	Sig. (2-tailed)	Sig. (2-tailed)	Sig. (2-tailed)	Sig. (2-tailed)	Sig. (2-tailed)	Sig. (2-tailed)	Sig. (2-tailed)	Sig. (2-tailed)	Sig. (2-tailed)	Sig. (2-tailed)	Sig. (2-tailed)	Sig. (2-tailed)	Sig. (2-tailed)
Log10_sBTLA		1.14E-06	1.18E-06	6.74E-02	2.67E-06	1.45E-01	1.10E-05	8.82E-08	3.71E-02	6.66E-03	7.01E-07	2.29E-04	3.16E-04	2.62E-03
Log10_sCD137	1.14E-06		1.86E-10	1.78E-01	5.03E-11	1.62E-01	1.84E-06	1.61E-05	5.66E-03	3.85E-03	1.28E-06	3.53E-07	3.28E-03	1.04E-02
Log10_sCD152	1.18E-06	1.86E-10		3.65E-02	4.02E-16	4.92E-02	2.27E-07	9.73E-08	4.97E-03	2.68E-03	1.51E-07	2.50E-09	9.43E-05	3.84E-04
Log10_sCD27	6.74E-02	1.78E-01	3.65E-02		2.23E-01	7.21E-01	2.74E-01	1.32E-01	5.40E-01	7.67E-01	2.81E-01	1.26E-02	6.69E-02	5.63E-05
Log10_sCD28	2.67E-06	5.03E-11	4.02E-16	2.23E-01		1.97E-01	4.29E-09	1.12E-07	7.30E-04	4.45E-04	1.95E-08	1.23E-10	3.83E-03	2.81E-03
Log10_sCD80	1.45E-01	1.62E-01	4.92E-02	7.21E-01	1.97E-01		2.27E-01	7.56E-01	9.20E-02	2.91E-02	5.33E-02	1.62E-01	1.47E-03	2.74E-02
Log10_sGITR	1.10E-05	1.84E-06	2.27E-07	2.74E-01	4.29E-09	2.27E-01		3.87E-07	1.27E-03	1.89E-02	2.29E-15	2.16E-06	6.48E-02	2.07E-02
Log10_sHVEM	8.82E-08	1.61E-05	9.73E-08	1.32E-01	1.12E-07	7.56E-01	3.87E-07		2.60E-03	7.99E-02	1.26E-06	5.30E-04	1.81E-02	5.20E-02
Log10_sIDO	3.71E-02	5.66E-03	4.97E-03	5.40E-01	7.30E-04	9.20E-02	1.27E-03	2.60E-03		6.68E-02	3.44E-03	6.58E-03	6.88E-02	1.25E-01
Log10_sLAG3	6.66E-03	3.85E-03	2.68E-03	7.67E-01	4.45E-04	2.91E-02	1.89E-02	7.99E-02	6.68E-02		5.49E-03	3.15E-03	3.29E-03	3.99E-02
Log10_sPD1	7.01E-07	1.28E-06	1.51E-07	2.81E-01	1.95E-08	5.33E-02	2.29E-15	1.26E-06	3.44E-03	5.49E-03		1.04E-05	2.16E-02	2.06E-02
Log10_sPDL1	2.29E-04	3.53E-07	2.50E-09	1.26E-02	1.23E-10	1.62E-01	2.16E-06	5.30E-04	6.58E-03	3.15E-03	1.04E-05		1.13E-02	4.48E-04
Log10_sPDL2	3.16E-04	3.28E-03	9.43E-05	6.69E-02	3.83E-03	1.47E-03	6.48E-02	1.81E-02	6.88E-02	3.29E-03	2.16E-02	1.13E-02		3.51E-05
Log10_sTIM3	2.62E-03	1.04E-02	3.84E-04	5.63E-05	2.81E-03	2.74E-02	2.07E-02	5.20E-02	1.25E-01	3.99E-02	2.06E-02	4.48E-04	3.51E-05	

	Log10_sBTLA	Log10_sCD137	Log10_sCD152	Log10_sCD27	Log10_sCD28	Log10_sCD80	Log10_sGITR	Log10_sHVEM	Log10_sIDO	Log10_sLAG3	Log10_sPD1	Log10_sPDL1	Log10_sPDL2	Log10_sTIM3
corr. coeff.	Pearson Correlation	Pearson Correlation	Pearson Correlation	Pearson Correlation	Pearson Correlation	Pearson Correlation	Pearson Correlation	Pearson Correlation	Pearson Correlation	Pearson Correlation	Pearson Correlation	Pearson Correlation	Pearson Correlation	Pearson Correlation
Log10_sBTLA	1.000	0.719	0.718	0.313	0.701	0.252	0.669	0.765	0.354	0.450	0.728	0.584	0.574	0.493
Log10_sCD137	0.719	1.000	0.844	0.233	0.857	0.242	0.709	0.660	0.458	0.476	0.717	0.741	0.483	0.428
Log10_sCD152	0.718	0.844	1.000	0.355	0.932	0.335	0.749	0.763	0.464	0.492	0.756	0.815	0.612	0.567
Log10_sCD27	0.313	0.233	0.355	1.000	0.211	-0.063	0.190	0.260	-0.107	-0.052	0.188	0.418	0.313	0.627
Log10_sCD28	0.701	0.857	0.932	0.211	1.000	0.223	0.808	0.761	0.544	0.562	0.788	0.848	0.476	0.490
Log10_sCD80	0.252	0.242	0.335	-0.063	0.223	1.000	0.210	0.054	0.289	0.369	0.329	0.242	0.517	0.373
Log10_sGITR	0.669	0.709	0.749	0.190	0.808	0.210	1.000	0.740	0.523	0.395	0.924	0.706	0.316	0.390
Log10_sHVEM	0.765	0.660	0.763	0.260	0.761	0.054	0.740	1.000	0.493	0.300	0.717	0.556	0.397	0.331
Log10_sIDO	0.354	0.458	0.464	-0.107	0.544	0.289	0.523	0.493	1.000	0.313	0.481	0.451	0.311	0.264
Log10_sLAG3	0.450	0.476	0.492	-0.052	0.562	0.369	0.395	0.300	0.313	1.000	0.459	0.485	0.483	0.349
Log10_sPD1	0.728	0.717	0.756	0.188	0.788	0.329	0.924	0.717	0.481	0.459	1.000	0.671	0.387	0.390
Log10_sPDL1	0.584	0.741	0.815	0.418	0.848	0.242	0.706	0.556	0.451	0.485	0.671	1.000	0.423	0.562
Log10_sPDL2	0.574	0.483	0.612	0.313	0.476	0.517	0.316	0.397	0.311	0.483	0.387	0.423	1.000	0.640
Log10_sTIM3	0.493	0.428	0.567	0.627	0.490	0.373	0.390	0.331	0.264	0.349	0.390	0.562	0.640	1.000

Supplementary table 3

Correlations

p-value	Log10_sBTLA Sig. (2-tailed)	Log10_sCD137 Sig. (2-tailed)	Log10_sCD152 Sig. (2-tailed)	Log10_sCD27 Sig. (2-tailed)	Log10_sCD28 Sig. (2-tailed)	Log10_sCD80 Sig. (2-tailed)	Log10_sGITR Sig. (2-tailed)	Log10_sHVEM Sig. (2-tailed)	Log10_sIDO Sig. (2-tailed)	Log10_sLAG3 Sig. (2-tailed)	Log10_sPD1 Sig. (2-tailed)	Log10_sPDL1 Sig. (2-tailed)	Log10_sPDL2 Sig. (2-tailed)	Log10_sTIM3 Sig. (2-tailed)
Log10_sBTLA		6.29E-05	3.65E-08	8.66E-01	9.38E-10	1.83E-05	4.28E-05	1.09E-06	2.67E-05	3.31E-04	7.84E-07	5.30E-03	1.56E-02	9.43E-01
Log10_sCD137	6.29E-05		7.53E-07	4.13E-01	5.51E-06	6.72E-07	2.27E-04	2.36E-03	3.34E-07	1.05E-05	1.88E-05	3.40E-02	5.34E-04	8.90E-01
Log10_sCD152	3.65E-08	7.53E-07		6.43E-01	7.94E-13	8.62E-07	8.57E-06	4.76E-05	2.72E-06	7.19E-05	9.11E-07	8.19E-03	1.53E-03	9.89E-01
Log10_sCD27	8.66E-01	4.13E-01	6.43E-01		7.04E-01	3.83E-01	7.61E-01	7.54E-01	5.91E-01	6.21E-01	6.77E-01	4.62E-02	4.08E-01	7.31E-04
Log10_sCD28	9.38E-10	5.51E-06	7.94E-13	7.04E-01		1.92E-06	1.59E-05	1.34E-05	7.16E-06	1.06E-04	4.20E-07	7.10E-03	3.81E-03	9.95E-01
Log10_sCD80	1.83E-05	6.72E-07	8.62E-07	3.83E-01	1.92E-06		8.81E-04	1.08E-03	3.89E-06	7.55E-07	3.62E-06	3.36E-02	5.08E-04	9.77E-01
Log10_sGITR	4.28E-05	2.27E-04	8.57E-06	7.61E-01	1.59E-05	8.81E-04		1.15E-03	2.95E-05	2.76E-03	1.31E-04	8.78E-04	4.17E-03	4.48E-01
Log10_sHVEM	1.09E-06	2.36E-03	4.76E-05	7.54E-01	1.34E-05	1.08E-03	1.15E-03		3.06E-03	8.48E-03	9.31E-05	4.12E-02	4.17E-02	6.83E-01
Log10_sIDO	2.67E-05	3.34E-07	2.72E-06	5.91E-01	7.16E-06	3.89E-06	2.95E-05	3.06E-03		6.32E-05	2.65E-06	1.41E-02	2.92E-03	8.14E-01
Log10_sLAG3	3.31E-04	1.05E-05	7.19E-05	6.21E-01	1.06E-04	7.55E-07	2.76E-03	8.48E-03	6.32E-05		1.96E-04	1.91E-02	1.33E-03	7.64E-01
Log10_sPD1	7.84E-07	1.88E-05	9.11E-07	6.77E-01	4.20E-07	3.62E-06	1.31E-04	9.31E-05	2.65E-06	1.96E-04		1.36E-02	4.95E-03	9.06E-01
Log10_sPDL1	5.30E-03	3.40E-02	8.19E-03	4.62E-02	7.10E-03	3.36E-02	8.78E-04	4.12E-02	1.41E-02	1.91E-02	1.36E-02		1.12E-01	2.29E-02
Log10_sPDL2	1.56E-02	5.34E-04	1.53E-03	4.08E-01	3.81E-03	5.08E-04	4.17E-03	4.17E-02	2.92E-03	1.33E-03	4.95E-03	1.12E-01		7.06E-01
Log10_sTIM3	9.43E-01	8.90E-01	9.89E-01	7.31E-04	9.95E-01	9.77E-01	4.48E-01	6.83E-01	8.14E-01	7.64E-01	9.06E-01	2.29E-02	7.06E-01	

corr. coeff.	Log10_sBTLA Pearson Correlation	Log10_sCD137 Pearson Correlation	Log10_sCD152 Pearson Correlation	Log10_sCD27 Pearson Correlation	Log10_sCD28 Pearson Correlation	Log10_sCD80 Pearson Correlation	Log10_sGITR Pearson Correlation	Log10_sHVEM Pearson Correlation	Log10_sIDO Pearson Correlation	Log10_sLAG3 Pearson Correlation	Log10_sPD1 Pearson Correlation	Log10_sPDL1 Pearson Correlation	Log10_sPDL2 Pearson Correlation	Log10_sTIM3 Pearson Correlation
Log10_sBTLA	1.000	0.883	0.971	-0.052	0.985	0.907	0.891	0.945	0.901	0.839	0.949	0.722	0.653	0.022
Log10_sCD137	0.883	1.000	0.949	-0.249	0.926	0.950	0.851	0.764	0.956	0.917	0.907	0.590	0.824	-0.042
Log10_sCD152	0.971	0.949	1.000	-0.142	0.996	0.948	0.920	0.889	0.935	0.880	0.947	0.696	0.783	0.004
Log10_sCD27	-0.052	-0.249	-0.142	1.000	-0.117	-0.264	0.094	-0.097	-0.165	-0.151	-0.128	0.561	-0.251	0.813
Log10_sCD28	0.985	0.926	0.996	-0.117	1.000	0.939	0.910	0.913	0.922	0.871	0.954	0.705	0.740	0.002
Log10_sCD80	0.907	0.950	0.948	-0.264	0.939	1.000	0.806	0.798	0.931	0.949	0.932	0.591	0.826	-0.009
Log10_sGITR	0.891	0.851	0.920	0.094	0.910	0.806	1.000	0.795	0.899	0.757	0.866	0.806	0.736	0.231
Log10_sHVEM	0.945	0.764	0.889	-0.097	0.913	0.798	0.795	1.000	0.751	0.694	0.874	0.572	0.571	-0.125
Log10_sIDO	0.901	0.956	0.935	-0.165	0.922	0.931	0.899	0.751	1.000	0.883	0.936	0.660	0.754	0.073
Log10_sLAG3	0.839	0.917	0.880	-0.151	0.871	0.949	0.757	0.694	0.883	1.000	0.855	0.638	0.789	0.093
Log10_sPD1	0.949	0.907	0.947	-0.128	0.954	0.932	0.866	0.874	0.936	0.855	1.000	0.663	0.726	0.036
Log10_sPDL1	0.722	0.590	0.696	0.561	0.705	0.591	0.806	0.572	0.660	0.638	0.663	1.000	0.461	0.623
Log10_sPDL2	0.653	0.824	0.783	-0.251	0.740	0.826	0.736	0.571	0.754	0.789	0.726	0.461	1.000	0.116
Log10_sTIM3	0.022	-0.042	0.004	0.813	0.002	-0.009	0.231	-0.125	0.073	0.093	0.036	0.623	0.116	1.000

Supplementary table 4

Coefficients (a,b)

Model	Collinearity Statistics	
	**VIF**	**Tolerance (1 / VIF)**
(Intercept)	---	---
Log10_sBTLA	6.068	0.165
Log10_sCD137	6.848	0.146
Log10_sCD152	29.904	0.033
Log10_sCD27	4.419	0.226
Log10_sCD28	34.009	0.029
Log10_sCD80	3.128	0.320
Log10_sGITR	6.595	0.152
Log10_sHVEM	9.329	0.107
Log10_sIDO	2.559	0.391
Log10_sLAG3	3.013	0.332
Log10_sPD1	6.372	0.157
Log10_sPDL1	6.879	0.145
Log10_sPDL2	4.059	0.246
Log10_sTIM3	5.670	0.176

a. Dependent Variable: Group (HC / ARC / SAH)

b. VIF > 5 indicates the presence of multicollinearity (the higher the stronger)

Supplementary table 5

Correlations

p-value	ChildPugh Score Sig. (2-tailed)	MELD Score Sig. (2-tailed)	Maddreys DF Sig. (2-tailed)	Creatinine (mmol/L) Sig. (2-tailed)	TotalBilirubin (mmol/L) Sig. (2-tailed)	AST Sig. (2-tailed)	GGT Sig. (2-tailed)	ALP Sig. (2-tailed)	Albumin (g/dL) Sig. (2-tailed)	INR Sig. (2-tailed)	Na (mmol/L) Sig. (2-tailed)	Platelets (billion/L) Sig. (2-tailed)	Total Leukocytes (billion/L) Sig. (2-tailed)	Neutrophils (billion/L) Sig. (2-tailed)	Lymphocytes (billion/L) Sig. (2-tailed)	Monocytes (billion/L) Sig. (2-tailed)	Neutrophil-to-Lymphocyte ratio (NLR) Sig. (2-tailed)	Monocyte-to-Lymphocyte ratio (MLR) Sig. (2-tailed)
Log_sTIM3	1.4E-02	1.5E-02	1.1E-01	3.7E-01	6.8E-01	9.3E-01	4.0E-01	6.1E-01	4.1E-02	6.5E-02	1.3E-02	9.6E-01	6.6E-01	6.4E-01	1.3E-01	1.7E-01	5.3E-01	2.0E-02
Log_sCD80	5.2E-04	3.7E-02	6.2E-01	1.3E-01	2.2E-01	4.4E-01	8.7E-01	9.9E-01	2.4E-05	7.5E-03	8.0E-01	8.2E-01	9.7E-01	7.0E-01	4.5E-01	7.0E-02	6.4E-01	8.2E-02
Log_sLAG3	4.6E-02	3.3E-01	6.7E-02	5.4E-01	9.9E-01	2.0E-01	1.6E-02	8.9E-02	1.0E-02	5.0E-05	5.2E-01	6.6E-01	1.0E-01	1.4E-01	1.6E-02	3.9E-01	9.4E-01	4.6E-01
Log_sPDL2	2.9E-03	1.6E-02	9.1E-01	2.8E-01	8.8E-01	7.3E-01	7.3E-01	2.3E-01	1.1E-01	4.9E-03	3.0E-01	9.4E-01	6.3E-02	8.8E-02	3.3E-02	7.6E-01	8.7E-01	1.0E-01
Log_sIDO	1.2E-01	7.9E-01	8.3E-01	3.2E-01	4.2E-01	4.2E-01	3.3E-01	1.0E-01	6.1E-01	8.3E-01	8.9E-01	3.3E-01	9.0E-01	9.2E-01	9.5E-01	2.3E-01	7.4E-01	3.6E-01
Log_sPDL1	9.6E-02	1.6E-01	8.1E-01	8.9E-01	9.5E-01	4.5E-01	1.1E-01	5.5E-02	4.4E-01	1.4E-01	9.6E-02	9.5E-01	6.2E-01	6.9E-01	9.6E-01	3.8E-01	9.0E-01	4.4E-01
Log_sCD27	4.9E-01	1.9E-02	6.7E-01	5.2E-02	3.7E-01	9.3E-01	5.9E-01	6.1E-01	6.8E-01	4.8E-01	1.8E-02	4.3E-01	4.2E-01	4.9E-01	9.4E-01	2.4E-01	1.6E-01	2.1E-01
Log_sPD1	8.0E-02	3.4E-01	9.2E-01	4.4E-01	7.8E-01	8.6E-01	1.3E-01	3.7E-01	1.8E-01	4.7E-02	3.5E-01	2.4E-01	8.8E-01	8.0E-01	7.2E-01	4.9E-01	9.1E-01	5.3E-01
Log_sGITR	1.6E-01	4.8E-01	8.7E-01	6.0E-01	7.5E-01	9.1E-01	4.7E-02	1.6E-01	4.6E-01	2.0E-01	3.3E-01	2.6E-01	6.6E-01	1.0E+00	4.3E-01	4.7E-01	6.5E-01	7.0E-01
Log_sCD28	1.3E-01	7.6E-01	8.3E-01	5.7E-01	6.2E-01	9.3E-01	6.3E-02	5.2E-02	5.3E-01	1.5E-01	5.3E-01	6.9E-01	1.8E-01	1.4E-01	8.3E-01	5.9E-01	3.2E-01	9.4E-01
Log_sHVEM	9.0E-01	7.5E-01	3.8E-01	7.6E-01	4.9E-01	5.3E-01	2.0E-01	2.3E-01	2.6E-01	7.3E-01	5.9E-01	6.4E-01	1.6E-01	2.2E-01	9.9E-01	5.1E-01	3.1E-01	9.0E-01
Log_sBTLA	7.2E-02	2.9E-01	4.0E-01	2.1E-01	8.1E-01	5.0E-01	1.7E-01	2.0E-01	6.5E-01	7.3E-02	9.7E-01	3.2E-01	6.9E-02	2.9E-01	2.6E-01	5.5E-01	9.7E-01	5.9E-01
Log_sCD152	7.7E-02	5.0E-01	5.1E-01	4.1E-01	7.5E-01	8.9E-01	2.5E-01	2.2E-01	4.0E-01	1.1E-01	4.0E-01	9.3E-01	2.0E-01	1.6E-01	9.6E-01	7.6E-01	2.0E-01	9.8E-01
Log_sCD137	7.3E-02	4.1E-01	6.2E-01	7.9E-01	9.4E-01	7.3E-01	3.1E-01	1.7E-01	5.4E-01	2.2E-01	5.1E-01	7.9E-01	2.5E-01	2.6E-01	8.4E-01	7.7E-01	4.3E-01	8.8E-01
Log_DLactate	1.0E-02	2.0E-03	1.0E+00	5.7E-01	4.0E-05	3.5E-01	9.6E-01	3.5E-01	8.4E-02	3.0E-01	3.0E-02	5.5E-01	3.6E-02	4.9E-02	3.2E-01	6.7E-03	1.6E-01	2.6E-01
Log_sCD163	7.3E-04	3.6E-02	1.5E-01	5.5E-01	4.8E-02	2.2E-01	7.9E-01	3.4E-01	4.7E-02	7.7E-02	2.3E-01	1.3E-01	6.6E-01	6.5E-01	6.4E-01	8.4E-01	5.8E-01	9.2E-01

p-value	Log_IL1a Sig. (2-tailed)	Log_IL1b Sig. (2-tailed)	Log_IL1ra Sig. (2-tailed)	Log_IL6 Sig. (2-tailed)	Log_IL8 Sig. (2-tailed)	Log_IL10 Sig. (2-tailed)	Log_IL18 Sig. (2-tailed)	Log_IL33 Sig. (2-tailed)	Log_IFNg Sig. (2-tailed)	Log_TNFa Sig. (2-tailed)	Log_DLactate Sig. (2-tailed)	Log_sCD163 Sig. (2-tailed)
Log_sTIM3	7.5E-02	3.0E-01	1.2E-01	3.6E-02	8.5E-01	7.3E-01	1.0E-01	1.5E-01	1.6E-01	1.0E-01	1.6E-02	3.0E-01
Log_sCD80	1.5E-01	1.0E-02	5.2E-02	2.8E-01	2.3E-01	9.7E-01	2.2E-02	1.4E-01	1.4E-02	1.2E-01	8.5E-03	2.3E-02
Log_sLAG3	5.3E-01	1.9E-01	1.7E-01	5.8E-02	1.8E-01	1.9E-01	8.2E-01	2.5E-01	1.3E-01	9.2E-01	7.0E-01	2.0E-01
Log_sPDL2	3.1E-01	3.5E-01	2.7E-01	1.7E-01	3.2E-01	8.0E-01	9.8E-02	7.9E-01	3.4E-01	3.2E-01	1.9E-01	5.2E-01
Log_sIDO	3.9E-02	1.9E-03	2.9E-03	1.2E-01	6.6E-01	5.3E-01	2.0E-03	7.8E-01	5.0E-01	7.9E-01	1.2E-01	8.8E-01
Log_sPDL1	8.9E-01	6.3E-01	9.3E-01	3.3E-01	3.3E-01	4.5E-01	3.5E-01	3.8E-01	3.7E-01	5.3E-01	1.4E-01	1.7E-01
Log_sCD27	5.9E-01	5.8E-01	9.3E-01	2.7E-01	4.6E-01	1.8E-01	6.9E-01	9.5E-01	7.8E-01	9.5E-01	1.8E-01	9.5E-01
Log_sPD1	7.1E-01	2.8E-01	9.5E-01	9.1E-01	4.1E-01	1.6E-01	2.3E-01	9.1E-01	8.0E-01	8.7E-01	5.1E-01	4.1E-01
Log_sGITR	5.9E-01	2.3E-01	8.5E-01	9.4E-01	6.5E-01	5.8E-01	2.6E-01	8.7E-01	7.4E-01	6.3E-01	2.6E-01	4.7E-01
Log_sCD28	8.5E-01	7.0E-01	9.4E-01	7.0E-01	4.7E-01	2.0E-01	3.0E-01	9.4E-01	7.4E-01	4.8E-01	3.7E-01	1.2E-01
Log_sHVEM	6.4E-01	3.2E-01	7.3E-01	3.5E-01	1.9E-01	5.4E-01	3.6E-01	7.7E-01	9.6E-01	1.8E-01	8.6E-01	4.1E-01
Log_sBTLA	2.5E-01	6.4E-01	9.2E-01	4.0E-01	1.5E-01	4.8E-01	4.0E-01	6.4E-01	7.7E-01	4.3E-01	7.6E-01	6.8E-01
Log_sCD152	9.6E-01	7.3E-01	9.3E-01	9.6E-01	4.7E-01	5.8E-01	5.0E-01	8.4E-01	9.5E-01	5.2E-01	2.9E-01	1.9E-01
Log_sCD137	9.2E-01	9.3E-01	7.7E-01	7.0E-01	5.2E-01	5.3E-01	6.1E-01	5.5E-01	7.2E-01	8.4E-01	4.4E-01	3.0E-01
Log_DLactate	1.9E-05	9.7E-04	2.7E-04	4.4E-02	1.1E-02	8.3E-03	3.9E-04	4.6E-03	6.8E-03	1.5E-02		
Log_sCD163	4.0E-01	7.8E-01	8.3E-01	1.4E-01	4.7E-02	1.7E-01	2.8E-01	3.2E-01	4.6E-01	2.2E-01		

corr. coeff.	ChildPugh Score Pearson Correlation	MELD Score Pearson Correlation	Maddreys DF Pearson Correlation	Creatinine (mmol/L) Pearson Correlation	TotalBilirubin (mmol/L) Pearson Correlation	AST Pearson Correlation	GGT Pearson Correlation	ALP Pearson Correlation	Albumin (g/dL) Pearson Correlation	INR Pearson Correlation	Na (mmol/L) Pearson Correlation	Platelets (billion/L) Pearson Correlation	Total Leukocytes (billion/L) Pearson Correlation	Neutrophils (billion/L) Pearson Correlation	Lymphocytes (billion/L) Pearson Correlation	Monocytes (billion/L) Pearson Correlation	Neutrophil-to-Lymphocyte ratio (NLR) Pearson Correlation	Monocyte-to-Lymphocyte ratio (MLR) Pearson Correlation
Log_sTIM3	0.443	0.409	-0.462	0.160	0.073	0.018	-0.167	-0.091	-0.347	0.315	-0.446	-0.010	-0.076	-0.081	-0.261	0.244	0.110	0.402
Log_sCD80	0.595	0.354	-0.153	-0.270	0.211	0.155	0.033	-0.003	-0.649	0.445	-0.049	0.042	-0.006	0.067	-0.132	0.320	0.083	0.307
Log_sLAG3	0.367	0.170	0.523	0.109	0.001	-0.254	-0.461	-0.296	-0.429	0.630	-0.121	-0.082	-0.283	-0.254	-0.403	-0.156	-0.014	0.133
Log_sPDL2	0.525	0.404	-0.034	0.195	-0.025	-0.069	-0.071	-0.211	-0.272	0.465	-0.195	-0.015	-0.318	-0.293	-0.362	-0.055	0.028	0.288
Log_sIDO	0.287	0.048	0.068	-0.180	0.142	0.162	-0.195	-0.284	-0.090	0.038	0.026	0.180	-0.022	0.017	-0.011	0.213	-0.059	0.165
Log_sPDL1	0.310	0.244	-0.075	-0.025	0.010	0.151	-0.317	-0.333	-0.136	0.256	-0.310	-0.012	-0.087	-0.069	-0.008	0.158	-0.022	0.139
Log_sCD27	0.132	0.394	-0.132	0.341	0.158	0.017	-0.108	0.090	0.072	0.125	-0.428	0.149	0.142	0.121	0.013	0.209	0.240	0.222
Log_sPD1	0.324	0.167	-0.031	-0.139	0.050	0.036	-0.298	-0.157	-0.234	0.339	-0.175	0.219	-0.027	0.045	0.063	0.125	-0.020	0.113
Log_sGITR	0.266	0.125	-0.052	-0.095	0.055	-0.022	-0.386	-0.244	-0.130	0.224	-0.183	0.210	-0.077	0.000	0.138	0.130	-0.078	0.071
Log_sCD28	0.281	0.053	-0.067	-0.103	-0.088	0.017	-0.362	-0.336	-0.109	0.251	-0.118	-0.074	-0.234	-0.255	-0.037	-0.097	-0.175	0.013
Log_sHVEM	0.024	-0.056	0.265	-0.055	-0.122	-0.127	-0.254	-0.212	0.194	0.061	0.102	0.088	-0.243	-0.213	-0.002	-0.120	-0.176	-0.022
Log_sBTLA	0.333	0.183	0.253	0.226	-0.042	-0.136	-0.273	-0.224	-0.079	0.307	-0.006	0.185	-0.311	-0.183	-0.196	-0.107	0.007	0.097
Log_sCD152	0.328	0.119	-0.199	-0.148	-0.055	0.029	-0.230	-0.218	-0.146	0.271	-0.161	-0.015	-0.221	-0.240	0.008	-0.056	-0.220	-0.004
Log_sCD137	0.332	0.145	0.152	0.047	-0.012	-0.070	-0.202	-0.242	-0.108	0.213	-0.125	0.049	-0.201	-0.195	0.036	-0.053	-0.139	0.028
Log_DLactate	0.468	0.511	0.002	0.103	0.644	0.187	-0.011	0.169	-0.300	0.184	-0.404	-0.114	0.361	0.340	0.174	0.470	0.247	0.206
Log_sCD163	0.591	0.360	-0.444	-0.109	0.342	0.242	0.054	0.171	-0.343	0.308	-0.231	-0.280	0.077	0.080	-0.083	0.037	0.098	0.018

corr. coeff.	Log_IL1a Pearson Correlation	Log_IL1b Pearson Correlation	Log_IL1ra Pearson Correlation	Log_IL6 Pearson Correlation	Log_IL8 Pearson Correlation	Log_IL10 Pearson Correlation	Log_IL18 Pearson Correlation	Log_IL33 Pearson Correlation	Log_IFNg Pearson Correlation	Log_TNFa Pearson Correlation	Log_DLactate Pearson Correlation	Log_sCD163 Pearson Correlation
Log_sTIM3	0.309	0.184	0.269	0.361	-0.033	0.063	0.286	0.255	0.245	0.285	0.411	0.182
Log_sCD80	0.253	0.434	0.337	0.191	0.210	-0.007	0.391	0.258	0.419	0.269	0.444	0.388
Log_sLAG3	-0.111	-0.231	-0.243	0.328	-0.233	-0.229	0.041	-0.202	-0.266	-0.018	0.069	0.224
Log_sPDL2	0.179	0.164	0.196	0.242	-0.174	0.044	0.288	0.048	0.169	0.175	0.230	0.115
Log_sIDO	0.356	0.514	0.495	-0.269	0.078	-0.112	0.510	0.050	0.121	0.048	0.271	0.028
Log_sPDL1	0.025	0.086	-0.016	0.172	-0.173	-0.134	0.165	-0.154	-0.159	-0.112	0.257	0.239
Log_sCD27	0.096	-0.098	-0.016	0.195	-0.130	0.234	-0.072	0.012	-0.049	0.011	0.234	-0.011
Log_sPD1	0.065	0.189	0.011	0.021	-0.146	-0.246	0.210	0.021	0.045	-0.028	0.118	0.146
Log_sGITR	0.097	0.210	0.034	-0.013	-0.080	-0.098	0.200	0.029	0.058	-0.085	0.198	0.127
Log_sCD28	0.033	0.068	-0.013	0.069	-0.129	-0.225	0.183	-0.013	-0.058	-0.124	0.160	0.272
Log_sHVEM	0.083	0.176	0.060	-0.165	-0.230	-0.108	0.162	-0.051	0.008	-0.233	0.032	-0.146
Log_sBTLA	0.205	0.083	0.018	0.148	-0.252	-0.124	0.149	-0.084	0.052	0.141	0.056	0.073
Log_sCD152	-0.009	0.062	-0.015	0.008	-0.130	-0.097	0.120	-0.035	0.010	-0.113	0.187	0.230
Log_sCD137	0.017	-0.015	-0.053	0.070	-0.115	-0.112	0.091	-0.107	-0.064	0.036	0.138	0.181
Log_DLactate	0.664	0.540	0.586	0.348	0.430	0.446	0.573	0.475	0.456	0.416		
Log_sCD163	0.148	0.051	0.038	0.258	0.343	-0.243	0.192	0.177	0.130	0.214		

Supplementary table 6

Correlations

p-value	Log10_IL1a Sig. (2-tailed)	Log10_IL1b Sig. (2-tailed)	Log10_IL1ra Sig. (2-tailed)	Log10_IL6 Sig. (2-tailed)	Log10_IL8 Sig. (2-tailed)	Log10_IL10 Sig. (2-tailed)	Log10_IL18 Sig. (2-tailed)	Log10_IL33 Sig. (2-tailed)	Log10_IFNg Sig. (2-tailed)	Log10_TNFa Sig. (2-tailed)
Log10_sBTLA	5.87E-01	na	4.60E-01	1.63E-01	1.93E-01	5.74E-01	5.70E-01	7.01E-01	na	3.90E-01
Log10_sCD137	9.35E-01	na	7.43E-01	5.28E-01	7.36E-01	4.05E-01	5.35E-01	4.20E-01	na	3.43E-01
Log10_sCD152	6.70E-01	na	6.49E-01	2.96E-01	2.47E-01	4.20E-01	7.03E-01	5.43E-01	na	3.63E-01
Log10_sCD27	8.91E-01	na	1.67E-01	8.55E-01	4.32E-01	8.52E-01	4.13E-01	6.08E-01	na	8.43E-01
Log10_sCD28	6.59E-01	na	5.73E-01	2.47E-01	1.85E-01	4.85E-01	6.92E-01	6.07E-01	na	4.10E-01
Log10_sCD80	8.40E-01	na	4.68E-01	5.00E-01	3.09E-01	6.22E-01	3.59E-01	2.87E-01	na	5.20E-01
Log10_sGITR	6.04E-01	na	8.13E-01	1.88E-01	2.93E-01	2.56E-01	7.05E-01	5.08E-01	na	3.30E-01
Log10_sHVEM	4.31E-01	na	1.42E-01	1.62E-01	1.48E-01	3.32E-01	9.83E-01	9.79E-01	na	2.83E-01
Log10_sIDO	9.32E-01	na	9.43E-01	1.70E-01	5.88E-01	6.84E-01	3.14E-01	3.07E-01	na	4.02E-01
Log10_sLAG3	3.40E-01	na	7.41E-01	8.99E-01	4.26E-01	5.75E-01	1.82E-01	2.24E-01	na	9.58E-01
Log10_sPD1	7.74E-01	na	5.58E-01	1.34E-01	1.86E-01	6.00E-01	5.21E-01	3.54E-01	na	2.51E-01
Log10_sPDL1	7.69E-01	na	3.42E-01	5.04E-01	1.61E-01	6.65E-01	3.27E-01	5.54E-01	na	5.18E-01
Log10_sPDL2	7.11E-01	na	9.80E-01	8.16E-01	3.69E-01	6.16E-02	5.88E-01	1.18E-01	na	3.93E-01
Log10_sTIM3	6.81E-01	na	7.26E-02	6.59E-01	3.42E-01	9.39E-01	8.39E-02	2.17E-01	na	9.48E-01

corr. coeff.	Log10_IL1a Pearson Correlation	Log10_IL1b Pearson Correlation	Log10_IL1ra Pearson Correlation	Log10_IL6 Pearson Correlation	Log10_IL8 Pearson Correlation	Log10_IL10 Pearson Correlation	Log10_IL18 Pearson Correlation	Log10_IL33 Pearson Correlation	Log10_IFNg Pearson Correlation	Log10_TNFa Pearson Correlation
Log10_sBTLA	-0.166	na	-0.225	-0.411	0.385	-0.172	0.174	-0.118	na	-0.260
Log10_sCD137	0.025	na	-0.101	-0.193	0.104	-0.252	0.190	-0.245	na	-0.286
Log10_sCD152	-0.131	na	-0.140	-0.314	0.346	-0.245	0.117	-0.186	na	-0.275
Log10_sCD27	0.042	na	0.408	0.056	0.239	-0.058	0.249	0.157	na	-0.061
Log10_sCD28	-0.136	na	-0.172	-0.346	0.393	-0.213	0.122	-0.158	na	-0.250
Log10_sCD80	0.062	na	-0.221	-0.206	0.306	-0.151	0.277	-0.320	na	-0.197
Log10_sGITR	-0.159	na	0.073	-0.390	0.316	-0.340	0.116	-0.202	na	-0.294
Log10_sHVEM	-0.239	na	-0.430	-0.412	0.424	-0.292	0.006	0.008	na	-0.322
Log10_sIDO	-0.026	na	-0.022	-0.405	0.166	-0.125	0.303	-0.307	na	-0.254
Log10_sLAG3	0.288	na	-0.102	-0.039	0.242	-0.172	0.395	-0.362	na	-0.016
Log10_sPD1	-0.088	na	-0.179	-0.439	0.392	-0.161	0.196	-0.280	na	-0.343
Log10_sPDL1	-0.090	na	0.287	-0.204	0.413	-0.133	0.295	-0.181	na	-0.198
Log10_sPDL2	0.114	na	0.008	0.072	0.272	-0.532	0.166	-0.455	na	-0.259
Log10_sTIM3	0.126	na	0.514	0.135	0.287	-0.023	0.497	-0.368	na	-0.020

Supplementary table 7

Supplementary figure legends.

Supplementary figure 1. Pro-inflammatory and anti-inflammatory cytokines in ALD patients. (A) Significance plot summarising cytokine measurements in HC, ARC and SAH; cytokine measurements: standardised z-scores; significance: $-Log_{10}$(p-value); red continuous line: BH significance threshold; red dotted line: p=0.05; black bars: significantly different cytokines; grey bars: non-significant cytokines. Additionally, individual boxplots for cytokine measurements in HC, ARC and SAH; KWp: raw Kruskal-Wallis p-value; BHq: FDR-adjusted q-value; p: significant multiple comparisons with Dunn's correction. Boxplots (median, IQR, ±Tukey's whiskers/outliers) ordered by decreasing statistical significance.

Supplementary figure 2. Soluble-CR levels in whole blood from four blood compartments in ARC patients. No differences in soluble-CR levels in plasma obtained from four anatomical compartments in 20 ARC patients undergoing TIPS; measurements are matched by subject (Friedman's paired test).

Supplementary figure 3. Soluble-CRs are not released from the liver upon acute ethanol (EtOH) exposure. (A) Lack of soluble-CR production in PCLS treated with ethanol 250 mM for 24 hours (Mann-Whitney test. Each dot represents one slice; each colour represents one subject). Boxplots (median, IQR, ±full range). (B) Lack of cellular toxicity by EtOH, measured as total cell death or % apoptotic cell death by cytokeratin (CK)18 fragments ELISA. Each dot represents one slice; red line: median. (C) Haematoxylin and eosin staining of PCLS, showing maintenance of tissue architecture after EtOH treatment.

Supplementary figure 4. Soluble-CR levels in whole blood upon acute bacterial challenge. Unchanged soluble-CR levels in E. coli-pulsed whole blood (Mixed Model analysis, with significant fixed effect by subject group but not by stimulation).

Supplementary figure 5. Representative FACS gating. Representative FACS plots showing the sequential gating for the measurement of membrane-TIM3 expression on T-cells (CD4/CD8), NK-cells (CD56hi/CD56lo), B-cells and monocytes.

Supplementary figure 6. Expression of membrane-TIM3 on immune subsets and modulation by E. coli. (A) membrane-TIM3 % at baseline (day 1, unstimulated) on T-cells (CD4/CD8), NK-cells (CD56hi/CD56lo), B-cells and monocytes (Kruskal-Wallis test with Dunn's multiple comparison correction). (B) Modulation of membrane-TIM3 % during a 7-day E. coli-stimulated culture; assessments performed at days 1-4-7; data expressed as E. coli fold ratio vs unstimulated medium (Mixed Model analysis, with fixed effect comparisons by group). Boxplots: median, IQR, ±Tukey's whiskers/outliers.

Supplementary figure legends.

Supplementary figure 7. Plasma soluble-TIM3 correlates with membrane-TIM3 expression on T-cells and NK-cells, and with their response to E. coli stimulation. Plasma soluble-TIM3 levels are positively correlated with basal (unstimulated) membrane-TIM3 expression in T-cells and NK-cells. However, plasma soluble-TIM3 levels correlate negatively with membrane-TIM3 upregulation response during antibacterial challenge. Each dot represents one subject; red continuous line: linear regression line; red dotted lines: 95% CI bands for the linear regression line. Correlations evaluated by Spearman's correlation analysis.

Supplementary figure 8. Galectin-9 blockade is not sufficient to rescue anti-bacterial responses in SAH patients. Specific blocking of Galectin-9 in PBMC cultures from SAH patients (n=4) with a neutralising monoclonal antibody was not sufficient to rescue the production of E. coli-stimulated antibacterial T-cell IFNγ (A) or anti-inflammatory T-cell IL-10 (C). Its effects on the production of pro-inflammatory IL-17A by CD8 T cells (B) and anti-inflammatory IL-10 by monocytes (D) were marginal and almost non-significant. Shading identifies different culture conditions as specified along each graph's x-axis. Boxplots: median, IQR, ±Tukey's whiskers/outliers.

Supplementary table legends.

Supplementary table 1. Antibodies used for flow cytometry. All the markers are indicated with their respective fluorochrome, antibody clone, physical excitation/emission lines and manufacturer's details.

Supplementary table 2. Soluble-CR levels measured in HC, ARC and SAH plasma.

Supplementary table 3. Intercorrelation matrix of soluble-CRs in ALD patients. The table lists the p-values and correlation coefficients calculated by Pearson's correlation analysis.

Supplementary table 4. Intercorrelation matrix of soluble-CRs in healthy controls. The table lists the p-values and correlation coefficients calculated by Pearson's correlation analysis.

Supplementary table 5. Variance Inflation factor analysis for plasma soluble-CR measurements in ALD patients and HC. The table lists the 'variance inflation factor' for each soluble-CR, a measurement of multivariate collinearity/correlation. Values above 5 indicate the presence of collinearity.

Supplementary table 6. Correlation analysis for plasma soluble-CR levels, cytokine measurements, clinical parameters and surrogate markers of bacterial translocation (D-lactate and soluble-CD163) in ALD patients. The table lists the p-values and correlation coefficients calculated by Pearson's correlation analysis.

Supplementary table 7. Lack of correlation between plasma soluble-CR levels and pro-/anti-inflammatory cytokines in healthy controls. The table lists the p-values and correlation coefficients calculated by Pearson's correlation analysis.
